# Influence of perinatal and childhood exposure to tobacco and mercury in children’s gut microbiota

**DOI:** 10.3389/fmicb.2023.1258988

**Published:** 2024-01-05

**Authors:** Sonia Pérez-Castro, Giuseppe D’Auria, Maria Llambrich, Sílvia Fernández-Barrés, Maria-Jose Lopez-Espinosa, Sabrina Llop, Benito Regueiro, Mariona Bustamante, M. Pilar Francino, Martine Vrijheid, Léa Maitre

**Affiliations:** ^1^Microbiology Department, Complexo Hospitalario Universitario de Vigo (CHUVI), Vigo, Spain; ^2^Microbiology and Infectology Research Group, Galicia Sur Health Research Institute (IIS Galicia Sur), SERGAS-UVIGO, Vigo, Spain; ^3^Spanish Consortium for Research on Epidemiology and Public Health (CIBERESP), Madrid, Spain; ^4^Sequencing and Bioinformatics Service, Fundació per al Foment de la Investigació Sanitària i Biomédica de la Comunitat Valenciana (FISABIO), Valencia, Spain; ^5^ISGlobal, Barcelona, Spain; ^6^Universitat Pompeu Fabra (UPF), Barcelona, Spain; ^7^Epidemiology and Environmental Health Joint Research Unit, Foundation for the Promotion of Health and Biomedical Research in the Valencian Region, FISABIO–Universitat Jaume I–Universitat de València, Valencia, Spain; ^8^Faculty of Nursing and Chiropody, University of Valencia, Valencia, Spain; ^9^Àrea de Genòmica i Salut, Fundació per al Foment de la Investigació Sanitària i Biomèdica de la Comunitat Valenciana (FISABIO)-Salut Pública, Valencia, Spain

**Keywords:** second-hand smoke, mercury, children, gut microbiota, birth cohort, 16S rRNA, diet, smoking during pregnancy

## Abstract

**Background:**

Early life determinants of the development of gut microbiome composition in infants have been widely investigated; however, if early life pollutant exposures, such as tobacco or mercury, have a persistent influence on the gut microbial community, its stabilization at later childhood remains largely unknown.

**Objective:**

In this exposome-wide study, we aimed at identifying the contribution of exposure to tobacco and mercury from the prenatal period to childhood, to individual differences in the fecal microbiome composition of 7-year-old children, considering co-exposure to a width of established lifestyle and clinical determinants.

**Methods:**

Gut microbiome was studied by 16S rRNA amplicon sequencing in 151 children at the genus level. Exposure to tobacco was quantified during pregnancy through questionnaire (active tobacco consumption, second-hand smoking -SHS) and biomonitoring (urinary cotinine) at 4 years (urinary cotinine, SHS) and 7 years (SHS). Exposure to mercury was quantified during pregnancy (cord blood) and at 4 years (hair). Forty nine other potential environmental determinants (12 at pregnancy/birth/infancy, 15 at 4 years and 22 at 7 years, such as diet, demographics, quality of living/social environment, and clinical records) were registered. We used multiple models to determine microbiome associations with pollutants including multi-determinant multivariate analysis of variance and linear correlations (wUnifrac, Bray-Curtis and Aitchison ß-diversity distances), single-pollutant permutational multivariate analysis of variance adjusting for co-variates (Aitchison), and multivariable association model with single taxa (MaAsLin2; genus). Sensitivity analysis was performed including genetic data in a subset of 107 children.

**Results:**

Active smoking in pregnancy was systematically associated with microbiome composition and ß-diversity (*R*^2^ 2–4%, *p* < 0.05, Aitchison), independently of other co-determinants. However, in the adjusted single pollutant models (PERMANOVA), we did not find any significant association. An increased relative abundance of *Dorea* and decreased relative abundance of *Akkermansia* were associated with smoking during pregnancy (*q* < 0.05).

**Discussion:**

Our findings suggest a long-term sustainable effect of prenatal tobacco exposure on the children’s gut microbiota. This effect was not found for mercury exposure or tobacco exposure during childhood. Assessing the role of these exposures on the children’s microbiota, considering multiple environmental factors, should be further investigated.

## 1 Introduction

Exposure to tobacco smoke and mercury during critical developmental stages poses significant threats to children’s health (Peterson and Hecht, 2017). During pregnancy, tobacco smoke is responsible for adverse outcomes in the offspring, such as impaired fetal development, heightened risk of obesity and cardiovascular diseases, compromised respiratory function, and increased vulnerability to conditions, such as asthma. Second-hand tobacco smoke (SHS) exposure during childhood has been related to asthma and other upper and lower respiratory illnesses, middle ear disease, and even syndrome of sudden infant death. For these reasons, there are strong recommendations to reduce children’s tobacco exposure (Jenssen et al., 2023). Mercury exposure, particularly in children, poses risks to neurological, nephrological, and immunological functions (Ruggieri et al., 2017), with potential consequences for neurodevelopment, cognitive abilities, and motor skills (US EPA O, 2015; Nutrition C for FS and A, 2023).

The intestine, host to a myriad of microbes, stands as a critical organ for metabolizing toxicants (Claus et al., 2016; National Academies of Sciences Engineering and Medicine, 2018; Koontz et al., 2019). While the diversity, structure, and functional potential of the intestinal microbiome are anticipated to undergo alterations due to toxicant exposure such as tobacco and mercury (Jin et al., 1987; Rosenfeld, 2017; Tsiaoussis et al., 2019), there is a notable scarcity of studies exploring these dynamics in humans, particularly in the context of children. Alterations of the gut microbiota in several animal models following exposure to mercury have been described including enrichment in microorganisms resistant to mercury and multiple antibiotics (Lloyd et al., 2016). The main mechanisms by which smoking affects the gut microbiota include the following: raising the pH of the intestinal environment, inducing chronic low-grade inflammation or inflammation-related diseases by inducing an increased abundance of proinflammatory bacteria, and promoting oxidative stress. Limited evidence on the impact of maternal smoking on the infant gut microbiota and its association with child overweight has been published (McLean et al., 2019).

Investigating the intricate associations between these exposures and the child gut microbiota is a complex task, which is exacerbated by the often-overlooked influence of various determinants, including lifestyle factors, and genetic predispositions. Previous reports emphasized the importance of day care attendance, household exposures (such as siblings or pets), or adherence to Mediterranean diet (Stewart et al., 2018; Barcik et al., 2020; Amir et al., 2022; Christensen et al., 2022; de Franchis et al., 2022). In addition, child body mass index (BMI) has been associated with their gut microbiota (Bai et al., 2019; Vander Wyst et al., 2021), whereas the risk of asthma could be related to the microbiome maturation along the first year of life (Stokholm et al., 2018). Additionally, mother BMI during pregnancy was also considered for its possible role as a risk factor for overweight and obesity across childhood (Voerman et al., 2019).

The human gut microbiota undergoes its most significant changes in infancy, from birth to the age of 3 years. Afterward, it is generally considered to be relatively stable over time (De Filippo et al., 2010; Hollister et al., 2015; Nakayama et al., 2015; Lundgren et al., 2018; Stewart et al., 2018; Zhong et al., 2019; Mesa et al., 2020; Yee et al., 2020; Ronan et al., 2021; Xie et al., 2021; Wernroth et al., 2022). Few studies have analyzed the persistent influence of early-life determinants on the composition of the gut microbiota along childhood, which is defined as the period of 3–11 years. It was suggested that breastfeeding, antibiotics use, having pets at home, older siblings, and DIETARY LIFESTYLE BY dietary intake (such as fiber and total fat consumption) could influence the child microbiome (Laursen et al., 2015; Nielsen et al., 2018; Savin et al., 2018; Wernroth et al., 2022; Panzer et al., 2023). In addition, the environmental pollutants are other possible determinants of child gut microbiota even less studied (Jin et al., 1987; National Academies of Sciences Engineering and Medicine, 2018; Iszatt et al., 2019; Koontz et al., 2019; Tsiaoussis et al., 2019).

Among all of these environmental pollutants, evidence of exposure to tobacco and mercury in children studied as part of the INMA cohorts in Spain highlights the importance of studying the impact of these pollutants on the gut microbiota of children. Based on the recent reports, levels exceeding the cotinine level equivalent to serum cutoff value of minor second-hand smoker (0.1 ng/mL) and the mercury level equivalent to the current US Environmental Protection Agency reference dose (5.8 μg/L of MeHg in whole blood) were detected. At 4 years old, INMA children were exposed to second-hand smoke (SHS) at home (21.6%) and elsewhere (47.1%) based on parental reports (Aurrekoetxea et al., 2016). In addition, 28.2% of children, as reported by parents to have no regular exposure to second-hand smoke (SHS), exhibited quantifiable urinary cotinine (UC) values. Among children from the INMA Valencia Cohort, a region close to the cohort studied in this article, 24% at birth and 19% at 4 years old exhibited mercury concentrations surpassing the threshold defined by the World Health Organization’s Provisional Tolerable Weekly Intake proposed by WHO (Llop et al., 2014) (i.e., 2.5 μg/g of body weight per week in cord blood and 1 μg/g in hair).

On the other hand, the role of host genetics in determining gut microbiome composition has been discussed (Hall et al., 2017; Rothschild et al., 2018). Recently, some microbiome-associated human genetic variants that could correlate with the relative abundance of microorganisms at the strain level were described (Markowitz et al., 2022). Interestingly, a functional FUT2 gene encodes alpha1,2-fucosyltransferase II that is responsible for the fucosylation of mucosal surfaces of the gut. It lately described the association of the lack of a functional FUT2 gene in children with infant microbial colonization and metabolic activity (Thorman et al., 2023).

In this study, we leveraged the in-depth longitudinal, phenotypic, and genotypic information available in children from the INMA-Sabadell birth cohort to address these research gaps. We evaluated the influence of tobacco smoke and mercury environmental exposures during pregnancy and childhood on diversity and genus abundance of the gut microbiome of 7-year-old children, considering other important early life determinants listed above. The wealth of data on the exposure assessment side and the gut microbiota profiling data generated makes this project a unique opportunity to investigate early life pollutant toxicity.

## 2 Materials and methods

### 2.1 Study population

A population-based birth cohort was established in the city of Sabadell (Catalonia, Spain) as part of the INMA Project (Guxens et al., 2012). Between July 2004 and July 2006, 657 pregnant women who visited the primary health center of Sabadell for an ultrasound in the 1st trimester were recruited. Inclusion criteria were: age at least 16 years, intention to give birth in the reference hospital, no problems in communication, singleton pregnancy, and no assisted conception. Informed consent was signed, and the study was approved by the ethics committee of the Institut Municipal d’Investigacio Medica (IMIM), Barcelona, Spain.

**Figure 1:**
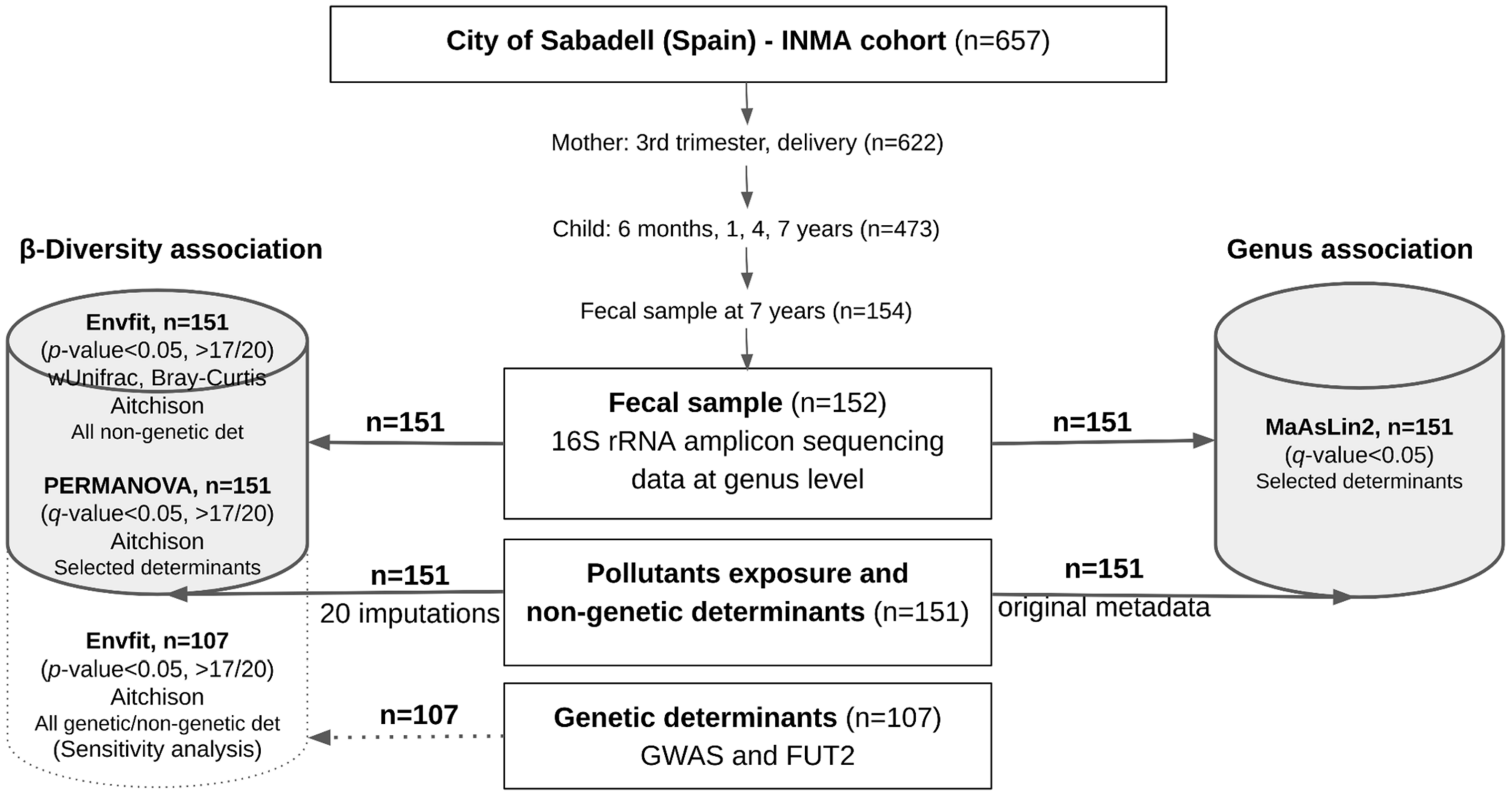
Graphic representation of the gut microbiome association analysis workflow in the Sabadell-INMA cohort (Sabadell, Catalonia, Spain). Data for gut microbiome at the genus level, exposure to pollutants (tobacco and mercury) and possible non-genetic determinants (clinical records, demographics, diet, and quality of living) were available for 151 children and were used in the main association analysis. For pollutant exposure and non-genetic determinants, missing data were imputed and included in the β-diversity association study, whereas the original data were included in the genus association study. When using imputations, an association was considered significant when a *p*-value <0.05 was observed in more than 17 out of 20 imputations. Sensitivity analysis was performed including genetic data of a subset of 107 children. A *p*-value or *q*-value (adjusted by confounders) <0.05 was considered, as appropriate. Full description of the statistical analysis is available in the materials and methods section.

Among 657 mother–infant pairs initially enrolled, 622 participated in the follow-up and conducted at the time of delivery. The mother–child pairs were later followed in the third trimester of pregnancy, at delivery, and at ages of 6 months and 1, 4, and 7 years (participation rate in the last follow-up, 76%). As part of the 7-year follow-up, 154 out of 473 children (32.6%) provided stool samples and were included in the present analysis. A total of 152 children yielded good quality microbiome sequencing data, but only fecal microbiome data from 151 children with the required determinant information were further analyzed. A graphic representation of the workflow is presented in Figure 1.

### 2.2 Determinants and pollutant exposures

**Figure 2:**
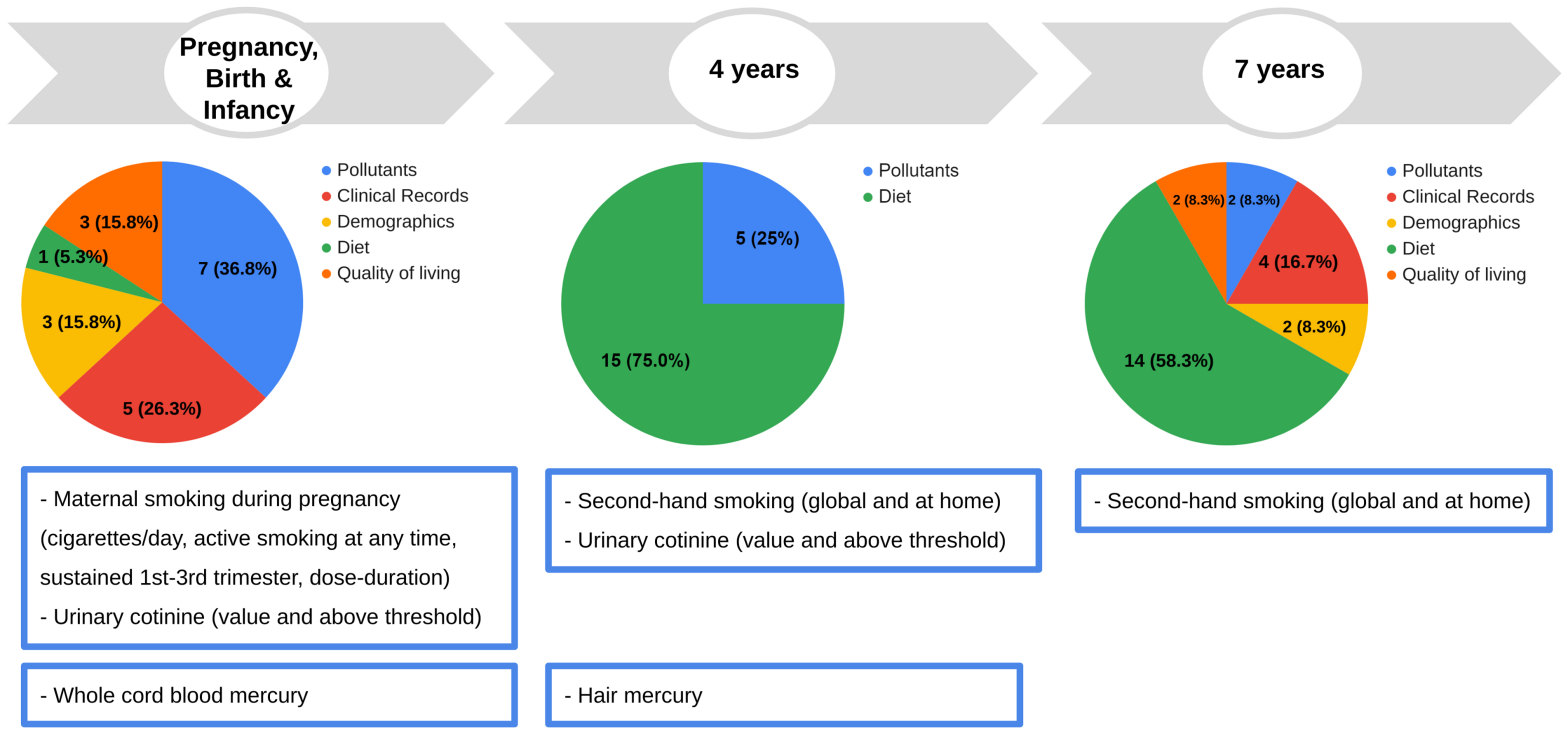
Early life and childhood determinants and pollutant exposures investigated in this study. In the pie chart, the number of variables each period and type are indicated. Data for each pollutant exposure and non-genetic determinant, or the imputed value in the case of missing value, were available for 151 children (see statistical analysis section). Original data of fish intake at 32 weeks of pregnancy were also available for 148 children. Original genetic data were available for a subset of 107 children. Full description of the variables is presented in Supplementary File 1; Table S1.

We considered all the variables collected as part of the INMA study from pregnancy to childhood and filtered them based on previous associations in the literature, prevalence, repeat measures (for diet and pollutants), missingness, and collinearity, resulting in 69 variables to be included in the association analyses (Figure 2), pertaining to six categories: pollutants (14 exposure variables), diet, demographics, quality of living/social environment, clinical records (49 non-genetic determinants), and genetics (6 genetic determinants). Full description of the variables is presented in Supplementary File 1; Table S1.

Some of them were related to birth and early life conditions, such as cesarean section delivery, predominant breastfeeding, siblings at birth, having pets at 14 months, attending day care center at age of 2 years, and maternal BMI during pre-pregnancy and pollutants. Others were related to factors at age of 4 or 7 years (number of servings/day of animal protein, dairy products, fruits and vegetables, fiber foods or sweet products, physical activity, asthma, vaccination, viral infections, and pollutants— mercury and tobacco smoke exposure sections) or specifically at 7 years of age [grade of adherence to Mediterranean diet measured by the Mediterranean Diet Quality Index (Serra-Majem et al., 2004)—KIDMED index, children BMI, and siblings]. General conditions such as sex, ethnic origin, and maternal education level were also collected.

Data for each non-genetic determinant and pollutant exposures were available for 151 children. In the case of missing values, the value was imputed, as explained in the statistical analysis section. Original genetic data were available for a subset of 107 children. The very specific dietary data obtained from the mothers (fish intake at 32 weeks of pregnancy), available for 148 children, was used only for adjustment in one specific mercury analysis, avoiding multiple imputation.

#### 2.2.1 Determinants

The individual determinants were reported by clinicians or available during face-to-face interview. Ethnic origin, maternal education (categorized as low-medium or high), and the presence of siblings at birth were obtained through questionnaires administered to the mothers during pregnancy, while the type of delivery (natural birth or cesarean delivery) was reported by the mother at birth. The fish intake at 32 weeks of pregnancy was obtained by face-to-face interview. Pet ownership, breastfeeding practices, and day care attendance at the age of 2 years were reported by the mothers in the questionnaires administered at 14 months and at 2 years, respectively. Predominant breastfeeding, defined as breast milk being the main source of nourishment accompanied by certain liquids, was categorized in: no predominant, only in the first 16 weeks, or for a longer period.

Dietary variables were extracted from a semi-quantitative food frequency questionnaire of 46 food items completed by parents at the age of 4 and/or 7 years. This questionnaire was adapted from a validated, child-specific food frequency questionnaire (Vioque et al., 2019). We evaluated animal protein foods, dairy products, fruits and vegetables, high-fiber foods, and sweet products as servings per day. Animal protein foods, dairy products, fruits and vegetables, high-fiber foods, and sweet products are expressed as g/day. We also evaluated a dietary score, representative of healthy eating, the Mediterranean Diet Quality Index for children and adolescents (KIDMED index), based on the principles of Mediterranean dietary patterns. The KIDMED index includes consumption of oil, fish, fruits, vegetables, cereals, nuts, pulses, pasta or rice, dairy products, and yogurt, which score positively and questions related to consumption of fast food, baked goods, sweets, and skipping breakfast which score negatively using a previously validated questionnaire (Štefan et al., 2017).

We measured children’s weight and height at the age of 7 years and calculated sex-specific and age-specific z-score body mass index, and overweight and obesity were defined following the World Health Organization standards (WHO, 2023). Obese and overweight children were analyzed together and compared with normal weight individuals. Ethnic origin, based on questionnaire data, was reclassified in the two groups: white European children (both parents white Europeans) and others (at least one parent not white European).

Genetic information was obtained from cord blood using the Omni1 Quad array from Illumina, as previously described (Bustamante et al., 2016). Information about the fucosyltransferase 2 (FUT2) gene was also available from the genetic data (Bustamante et al., 2016); children were classified as homozygous or non-homozygous for the “non-secretor” allele (AA) based on the polymorphism rs601338 G > A at the fucosyltransferase 2 (FUT2) gene. Finally, FUT2 rs601338 polymorphism was genotyped. The first five principal components of the genetic data were used in the sensitivity analysis.

#### 2.2.2 Tobacco smoke exposure

Tobacco smoke exposure was assessed through two complementary methods, by biomonitoring (cotinine measurements) and harmonized questionnaires, as previously evaluated (Vives-Usano et al., 2020). In brief, both were used for pregnant mothers and children of 4 years old, while only questionnaires were used for children of 7 years old. Active maternal smoking during pregnancy and exposure to SHS during pregnancy or childhood can be assessed through the combination of both methods.

Cotinine, the primary metabolite of nicotine, is a good biomarker of tobacco smoke exposure due to its medium half-life (16–18h) and its excretion in urine during the day. As urinary cotinine levels are highly correlated with plasma cotinine levels, they provide a valid measure of exposure to environmental tobacco smoke. Cotinine was determined in urine samples collected from mothers at week 32 of pregnancy and children of 4–5 years old. The laboratory method for urinary cotinine (UC) quantification was described in a previous study (Benowitz et al., 2009; Aurrekoetxea et al., 2013). Cotinine concentration was divided by total creatinine in urine to account for spot urine dilution. This exposure was also included as a dichotomized variable (exposed or not exposed) considering whether cotinine was detected in urine over the LOQ, indicating SHS exposure.

Tobacco smoke exposure was also collected through questionnaires during pregnancy and summarized as the mean number of cigarettes/day during pregnancy, maternal active smoking at any time during pregnancy, and sustained maternal smoking (first trimester and third trimester); maternal smoking dose-duration classified as: (Peterson and Hecht, 2017) unexposed, (Jenssen et al., 2023) only SHS and (Ruggieri et al., 2017) non-sustained smoker/sustained smoker at low dose (≤9 cigarettes per day, c/d)/sustained smoker at high dose (>9 c/d).

Childhood passive smoking exposure at 4 and 7 years was available through a harmonized questionnaire administered to the parents and summarized as secondhand smoke exposure at home (yes/no) and global second-hand smoke exposure (yes/no) (Vives-Usano et al., 2020).

#### 2.2.3 Mercury exposure

Mercury level was assessed by biomonitoring at two time points using different biological samples; whole cord blood samples were used to study the intra-utero exposure while hair was used to study the exposure of children of 4 years old.

The recommended samples to quantify the exposure to mercury are blood and urine. Detection of mercury in hair samples may be particularly useful to know the prior exposure to methyl-mercury. Hair from the initial 0.5 cm could be representative of the exposure before 1 to 3 weeks of collection. It is important to know that hair levels are not correlated with the blood levels or symptoms of toxicity by this pollutant. In addition, reports of hair levels related to exogenous contamination are not rare (Nuttall, 2006).

At birth, whole cord blood samples were collected using venipuncture of cord vessels before the placenta was delivered. Blood cord samples were processed, separated into aliquots of 1 mL, and then frozen at −80°C until analysis. Hair samples were collected from the occipital scalp when children were 4 years old, placed in a plastic bag, and stored at room temperature until analysis. The analyses of total mercury were carried out in the Public Health Laboratory of Alava (Basque Country, Spain) using, for both types of samples, thermal decomposition, amalgamation, and atomic absorption spectrometry. The laboratory method for cord blood and hair total mercury quantification was described in previous studies (Llop et al., 2012). The limit of quantification of the method (LOQ) was 2 μg/L for cord blood samples and 0.01 μg/g for hair samples.

### 2.3 Microbial 16S rRNA gene sequencing

#### 2.3.1 Sample processing and sequencing

Fecal samples collected in sterile containers were initially kept at home at 4°C and stored at −20°C within the first 2 days (1.5 h to 49 h) after collection. Genomic DNA extraction was performed using the NucliSENS^Ⓡ^ EasyMAG^Ⓡ^ instrument (bioMérieux). According to the manufacturer’s instructions, a portion of each fecal sample was collected with a sterile loop (approximately 40 mg) and lysed “on-board” in 2 mL of lysis buffer. The specific protocol B was used with 50 μL of magnetic silica. The total nucleic acids were recovered in 50 μL of elution buffer and stored at −20°C until use.

Microbial genomic DNA was used at a concentration of 5 ng/μl in 10 mMTris (pH 8.5). The library preparation of the 16S ribosomal RNA gene was performed according to the 16S Metagenomic Sequencing Library Preparation Illumina protocol (Part # 15044223 Rev. A, Illumina, San Diego, CA, USA). In brief, the V3-V4 hypervariable regions of the 16S rRNA gene were amplified using the forward primer 5’-TCGTCGGCAGCGTCAGATGTGTATAAGAGACAGCCTACGG GNGGCWGCAG-3′ and the reverse primer 5′-GTCTCGTGGGCT CGGAGATGTGTATAAGAGACAGGACTACHVGGGTATCTAATC C-3′, including the Illumina adapter overhang sequences. The Nextera XT DNA Library Preparation Kit (FC-131-1096, Illumina Inc, San Diego, USA) was used according to the manufacturer’s instructions. Amplification PCR conditions were: 95°C for 3 min, 25 cycles of 95°C for 30 s, 55°C for 30 s followed by 72°C for 30 s, and 72°C for 5 min. Positive and negative controls were included in the PCR. Index PCR conditions were: 95°C for 3 min, 8 cycles of 95°C for 30 s, 55°C for 30 s, and 72°C for 30 s followed by 72°C for 5 min.

After 16S rRNA gene amplification, 2 sets of 77 amplicons were multiplexed, and 1 μL of each amplicon pool was run on a Bioanalyzer DNA 1000 chip to verify amplicon size (~550 bp). After size verification, libraries were sequenced in an Illumina MiSeq platform, according to the manufacturer’s instructions, using two 2 × 300 cycle paired-end runs (MiSeq Reagent Kit v3 600 cycles) (MS-102-3001, Illumina Inc). Positive and negative controls were included in the PCR.

#### 2.3.2 Sequence data analysis

Reads were quality filtered and trimmed by the DADA2 R package version 1.12.1 and R version 3.5.1^1^ using truncating of forward reads set to 250 bp and truncating of reverse reads set to 200 bp. Quality control was performed by removing all the sequences with ambiguous bases and a quality score lower than 2 or with more than 2 or 5 expected errors in the forward or reverse sequences, respectively. Chimeras were removed. Denoised pairs of forward and reverse reads overlapping 20 nucleotides were merged, resulting in unique amplicon sequence variants (ASVs) (Callahan et al., 2016). For taxonomic analysis, the Naive Bayes classifier was pre-trained on the silva 138 database. Phylogenetic analysis was performed with FastTree (Price et al., 2009).

For the analysis of microbiota data, the Phyloseq R package version 1.26.1 was used. Only taxa with at least 10 counts present in 20% of samples were selected for analysis. ASVs were agglomerated to class, phylum, genus, and species levels.

### 2.4 Statistical analysis

All statistical analyses were performed using R software. Descriptive statistics of the original metadata were calculated as median (first quartile, third quartile) for continuous variables and as frequency (percentage) for categorical variables.

To avoid calculations of β-diversity with missing values, continuous variables were transformed and standardized to logarithm (log), interquartile range (IQR), or log of IQR, as appropriate. Then, missing values for exposures and determinants (excluding genetic, FUT2, and fish intake at 32 weeks of pregnancy data) were imputed using multiple imputations by chained equations to generate 20 imputed data sets (mice R package). Original data were used for genetic, FUT2, and fish intake at 32 weeks of pregnancy.

β-Diversity association analysis: In the first place, a multi-determinant analysis using the *envfit* function (*vegan* R package, 999 permutations) was employed to identify determinants, which were significantly correlated with the gut microbiome β-diversity. We included the following dissimilarity matrices at the genus level: wUnifrac, Bray–Curtis, and Aitchison (Gloor et al., 2017), which were calculated as the Euclidean distance of centered log ratio-transformed data after Bayesian Multiplicative replacement of count zeros. The *envfit* function performs multivariate analysis of variance (MANOVA) and linear correlations for categorical and continuous variables, respectively. It calculates the effect size (R^2^) and significance (*p*-value) of each variable, comparing the difference in the centroids of each group relative to the total variation. Mean and standard deviation of the R^2^ values obtained along the 20 imputations for 14 tobacco and mercury exposure variables and 49 non-genetic determinants were calculated. The mean of *p*-values was also calculated. An association was considered statistically significant if a *p*-value < 0.05 was obtained in 17 out of the 20 imputations. In addition, for a sensitivity analysis, genetic data were included in an additional *envfit* analysis considering the Aitchison distance in a subset of 107 children in the same conditions.

In the second place, additional analyses were performed for each pollutant exposure variable without adjustment (crude model) for potentially important determinants. Exposures to tobacco were adjusted by maternal education, maternal BMI, and the *envfit*-selected variables. Exposure to mercury during pregnancy was adjusted by fish intake at 32 weeks of pregnancy, maternal education, and the *envfit*-selected variables. Exposure to mercury at 4 years was adjusted by fish intake at 4 years, maternal education, and the *envfit*-selected variables. A permutational multivariate analysis of variance (PERMANOVA) test by distance matrices with Benjamini & Hochberg (BH) correction for multiple testing using the adonis function (*vegan* R package, 999 permutations) was used. Mean and standard deviation of R^2^ values obtained for the imputed datasets considering Aitchison distance were calculated. An association was considered significant when a BH-corrected *q*-value <0.05 was obtained for more than 17 out of the 20 imputed datasets. The mean of adjusted *q*-values was also calculated. The principal component analysis (PCA) plot with the clr-transformed features was obtained with the microViz R package for one imputation. Ellipses corresponding to 95% confidence intervals (CI) between categories were calculated.

Genus association study: At the genus level, taxa were normalized to relative abundance and regressed against individual tobacco and mercury exposures in a crude model, adjusting for the potentially important determinants selected for the PERMANOVA analysis, including the most important covariates selected by the *envfit* analysis, as described above (MaAsLin2 R package, by default conditions). Then, we selected individual exposures and their corresponding taxa from the raw MaAsLin2 output table and calculated the BH-corrected *q*-value. For the screening of significant taxa, a *q*-value <0.05 was considered statistically significant, as described previously (Shen et al., 2022).

## 3 Results

### 3.1 Characterizing the gut microbiome and pollutant exposures of early school-age children

After performing 16S rRNA sequencing and quality control, we acquired 34,444 to 285,423 sequencing reads per sample (mean ± SD: 83,143 ± 35,329 reads). Sequence data that support the findings of this study have been deposited in the EBI EMBL database (PRJEB69271). A total of 4,904 ASVs were detected, corresponding to 2 kingdoms, 13 phyla, 20 classes, 52 orders, 80 families, 228 genera, and 186 species. The core microbiota in Sabadell-INMA children at the phylum level, defined as phyla found in more than 85% of the samples, comprised 5 phyla with the following mean relative abundance: *Bacteroidota* 44.65%, *Firmicutes* 39.28%, *Verrucomicrobiota* 11.02%, *Actinobacteria* 2.74%, *Proteobacteria* 2.29%. The core microbiota at the genus level, defined as genera found in 95% of the samples, comprised 15 genera. The most abundant genera were *Bacteroides* (30.9%), *Akkermansia* (11.2%), *Alistipes* (11.1%), and *Faecalibacterium* (10.8%). We considered 207 ASVs for the association study corresponding to 2 kingdoms, 6 phyla, 9 classes, 20 orders, 30 families, 61 genera, and 58 species that were present in 20% of samples.

The description of the characteristics of the studied Sabadell-INMA birth cohort is shown in Supplementary File 2: Table S2. Exposures to pollutants (tobacco and mercury) were of particular interest. In the studied population, 46.6% of children were exposed to tobacco smoke *in-utero*, 30.3% during childhood based on the 4 year-old children data, and 17.9% along the full period (sustained exposure) based on questionnaires. The mean corrected cotinine levels were 224.1 μg/g for mothers and 17.5 μg/g for children. The median cotinine levels were 6.0 μg/g for mothers and 5.5 μg/g for children.

In this study, 65% of children had mercury levels in cord blood samples above 5.8 μg/L. At the age of 4 years, 38.4% of the children had hair mercury levels above 1 μg/g.

### 3.2 Associations of environmental determinants with gut microbiome ß-diversity

In the multi-determinant model, active smoking during pregnancy and SHS at 4 years based on detectable urinary cotinine levels (≥4 ng/mL) were associated with the β-diversity, explaining 2–4% of the variability (Aitchison distance, *p*-values: 0.0045 and 0.0322, respectively, Table 1). Having siblings at birth was also associated with the β-diversity (Bray–Curtis and Aitchison distances, *p*-values: 0.0214 and 0.0065, respectively). In the sensitivity analysis performed in a subset of 107 children considering also genetic data and Aitchison distance, active smoking during pregnancy and having siblings at birth remained associated with the β-diversity (*p*-values: 0.0016 and 0.0080, respectively). Genetic variables explained less than 5% of the β-diversity (Aitchison).

**Table 1:** Pollutants and determinants significantly associated with children gut microbiome β-diversity in a multi-determinant model (*envfit* analysis) encompassing 48 determinants.

β-diversity distance	Sample size	Variable	Mean *R*^2^ (%, SD)	Mean *p*-value
Bray–Curtis	*n* = 151	Having siblings at birth (yes/no)	2.48, 0.26	0.0214
Aitchison		Active tobacco smoking during pregnancy (yes/no)	3.81, 0.64	0.0045
		SHS based on urinary cotinine levels at 4 years (yes/no)	2.31, 0.18	0.0322
		Having siblings at birth (yes/no)	3.40, 0.13	0.0065
Aitchison	Sensitivity analysis (*n* = 107)	Active tobacco smoking during pregnancy (yes/no)	6.77, 0.09	0.0016
		Having siblings at birth (yes/no)	4.55, 0.21	0.0080

Mean and standard deviation of *R*^2^ and mean of the *p*-value obtained for 20 imputations. Sensitivity analysis considering also genetic data was performed in a subset of 107 children. An association was considered significant when a *p*-value < 0.05 was obtained in more than 17 out of the 20 imputed datasets. SHS, second-hand smoking.

Only the statistically significant results are shown in Table 1. All results for the analysis of childhood and prenatal determinants for the three distances and the sensitivity analysis are presented in Supplementary Table S3. We found no significant associations of β-diversity with mercury or other tobacco exposure variables.

**Figure 3:**
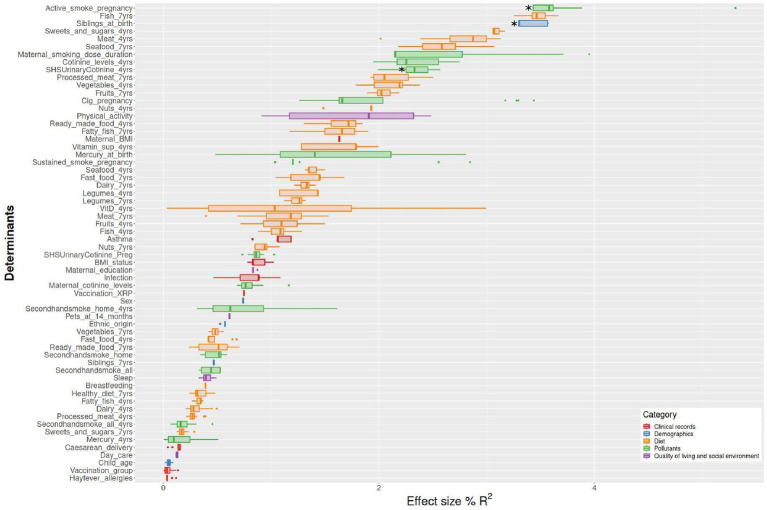
Effect size (*R*^2^) of microbiome β-diversity multiple determinants identified in the school-aged children of the Sabadell-INMA cohort. The analysis was based on the envfit function in the vegan R package. Factors are sorted according to their effect size and colored based on metadata category. Mean *R*^2^ was obtained for 20 imputed datasets considering, Aitchison distance (*n* = 151 children) Aitchison. Active smoking during pregnancy and SHS at 4 years based on urinary cotinine levels were associated with the β-diversity (Aitchison distance, *p*-value: 0.0045 and 0.0322, respectively). Having siblings at birth was also associated with the β-diversity (Aitchison distance, *p*-value: 0.0065).

The corresponding mean of *R*^2^ for 20 imputations obtained with the *envfit* analysis for all determinants and pollutant exposures are represented in Figure 3. All *R*^2^ and *p*-values are presented in Supplementary File 3: Table S3 and Supplementary File 4: Table S4.

**Figure 4:**
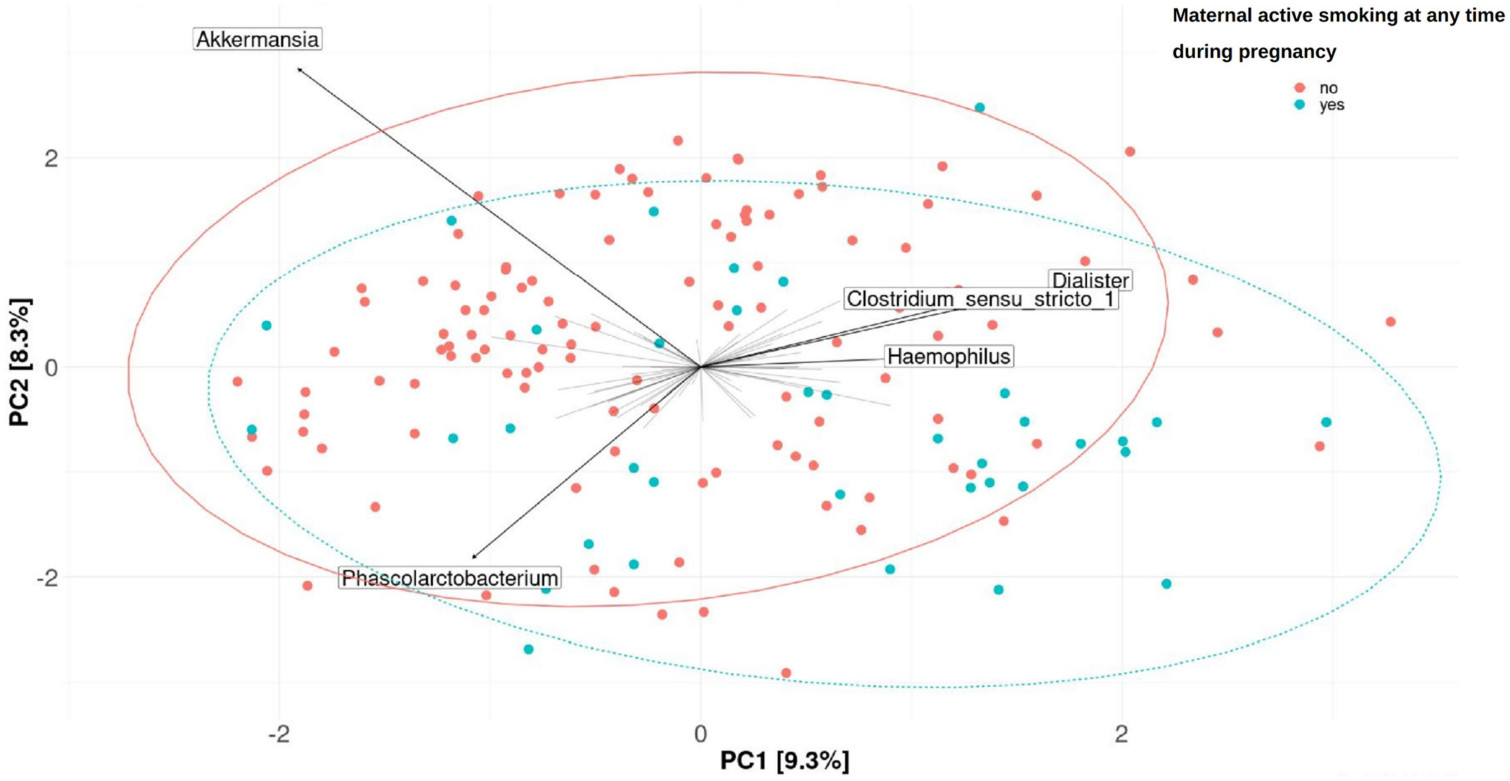
Fecal microbiome β-diversity from 7-year-old children, categorized by the exposure to active smoking of their mothers at any time during pregnancy. Plots of principal component analysis (PCA) with the clr-transformed features from children’s feces were calculated for one imputation set. The 95% confidence interval prediction ellipses were calculated for each category. Amplicon sequence variants (ASVs) were collapsed at the genus level. Each children’s microbial community is represented by a dot. Length of the arrows shows the strength of the association between each genus and the differences in microbiota composition: the arrow length is proportional to the variance explained by each specific genus; the arrow angle is correlated with the distribution of this variance between PC1 and PC2. *Akkermansia, Phascolarctobacterium, Dialister, Clostridium sensu-stricto*_1, and *Haemophilus* were driving the differences between children’s gut microbiomes. No significant association was found (PERMANOVA, *p*-value > 0.05 in the crude or adjusted univariate model).

**Figure 5:**
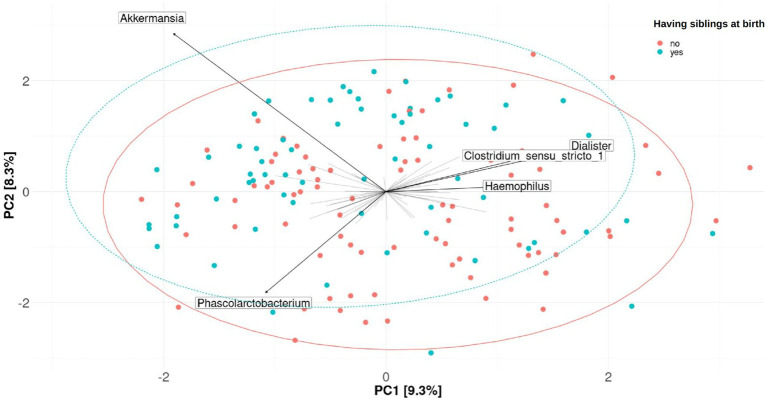
Fecal microbiome β-diversity from 7-year-old children, categorized by having siblings at birth. Plots of principal component analysis (PCA) with the clr-transformed features from children’s feces were calculated for one imputation set. The 95% confidence interval prediction ellipses were calculated for each category. Amplicon sequence variants (ASVs) were collapsed at the genus level. Each children’s microbial community is represented by a dot. Length of the arrows shows the strength of the association between each genus and the differences in microbiota composition: the arrow length is proportional to the variance explained by each specific genus; the arrow angle is correlated with the distribution of this variance between PC1 and PC2. *Akkermansia, Phascolarctobacterium, Dialister, Clostridium sensu-stricto*_1, and *Haemophilus* were driving the differences between children’s gut microbiomes. No significant association was found (PERMANOVA, *p*-value > 0.05 in the crude or adjusted univariate model).

On the other hand, β-diversity measured by the Aitchison distance was not significantly associated with the 12 tobacco exposure variables or the 2 mercury exposure variables included in this study in the single pollutant model (PERMANOVA). An unadjusted model was considered and a model adjusting by the covariates above-listed (see Material and methods section), considering “having siblings at birth” as the covariate selected by the envfit analysis. The results obtained for each of the 20 imputations and both models are shown in Supplementary File 5: Table S5. Plots of PCA with the clr-transformed features are shown by the exposure to active smoking of their mothers at any time during pregnancy and having siblings at birth in Figures 4 and 5, respectively.

### 3.3 Associations of exposure to tobacco and mercury with microbiome taxa (genus)

**Figure 6:**
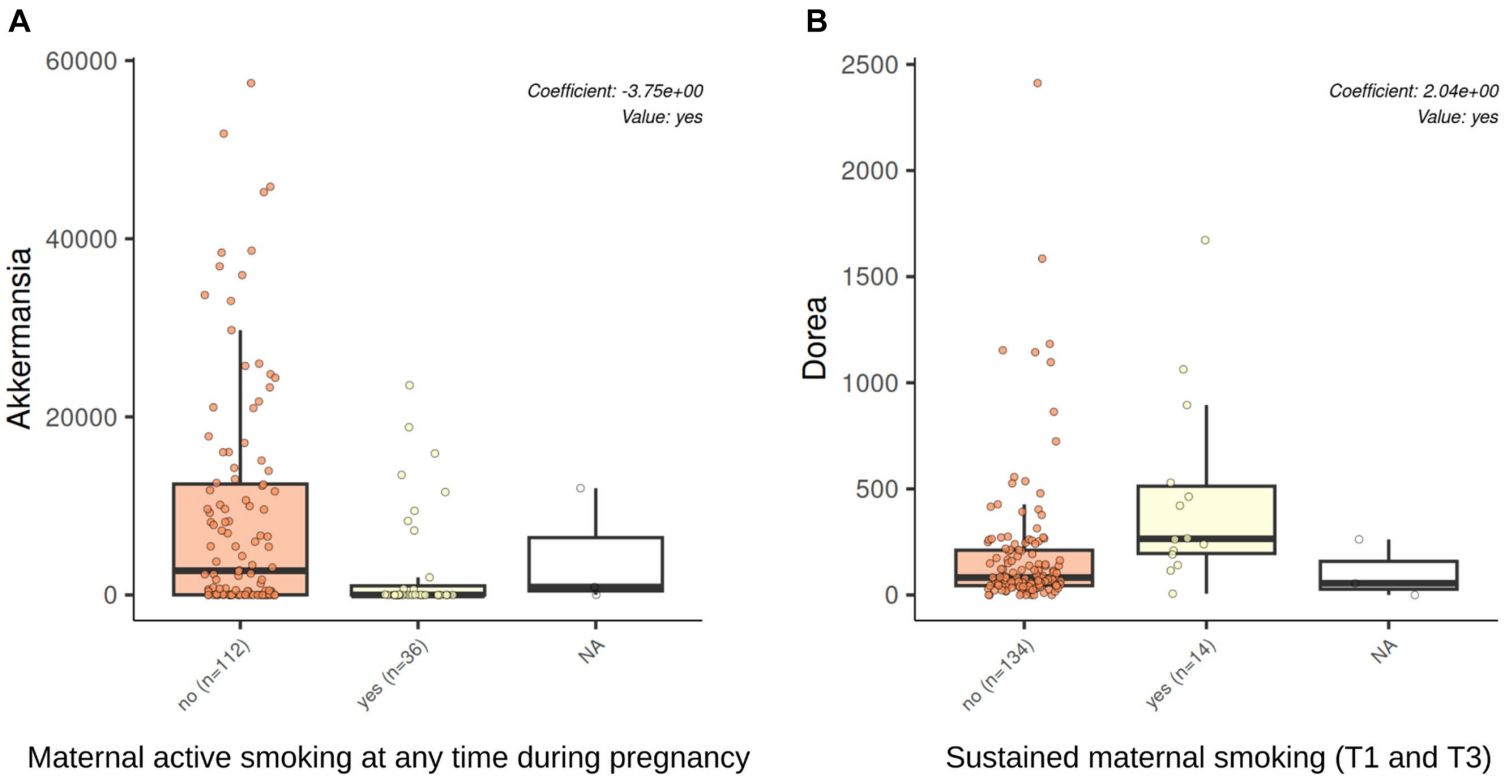
*Akkermansia* and *Dorea* abundances were associated with tobacco exposure through active smoking during pregnancy. The results of genus association with the multivariable association model (MaAsLin2) analysis in a model adjusted by maternal education, maternal BMI, and having siblings at birth. Active smoking at any time during pregnancy was associated with a decrease in the relative abundance of *Akkermansia* (*q*-value 0.005, BH adjusted by genus). Sustained maternal smoking from the first to the third trimester of pregnancy was associated with an increased relative abundance of *Dorea* (*q*-value 0.01, BH adjusted by genus).

Tobacco smoking during pregnancy was associated with the microbiota at 7 years of age at the genus level. The significant results are shown in Table 2. Active smoking at any time during pregnancy was associated with a decrease in the relative abundance of *Akkermansia* both in the unadjusted and the adjusted models (coef = −3.76, *q*-value 0.004, coef = −3.75, *q*-value 0.005, respectively). Sustained maternal smoking from first to third trimester of pregnancy was associated with an increased relative abundance of *Dorea* both in the unadjusted and adjusted models (coef = 2.01, *q*-value 0.006, coef = 2.04, *q*-value 0.01, respectively). Figure 6 shows the changes in the abundance of *Akkermansia* and *Dorea* associated with tobacco smoking during pregnancy considering the adjusted model. In addition, maternal smoking categorized as non-sustained, low dose, or high dose was associated with an increased relative abundance of *Dorea* (coef = 1.79, *q*-value = 0.03), although only in the unadjusted model. Mercury or tobacco exposure from pregnancy to childhood measured by other variables considered in this study were not associated with significant changes in the abundance of genus in the children’s gut microbiome at 7 years of age. The best 10 results (genus) with the smallest *q*-values obtained by the MaAsLin2 analysis are shown in Supplementary material 6: Supplementary Table 6.

**Table 2:** Association of perinatal tobacco exposure with child gut microbiome at the genus level in the multivariable association model (MaAsLin2 analysis).

Genus	Metadata	Value	coef	stderr	pval	qByGenus	Model
*Akkermansia*	Maternal active smoking at any time during pregnancy	Yes	−3.7643	0.9188	0.0001	0.0042	Unadjusted
*Dorea*	Sustained maternal smoking (T1 and T3)	Yes	2.0100	0.5049	0.0001	0.0065	Unadjusted
*Dorea*	Maternal smoking dose-duration	Non-sustained/low/high	1.7873	0.4788	0.0003	0.0326	Unadjusted
*Akkermansia*	Maternal active smoking at any time during pregnancy	Yes	−3.7498	0.9279	0.0001	0.0052	Adjusted
*Dorea*	Sustained maternal smoking (T1 and T3)	Yes	2.0427	0.5279	0.0002	0.0100	Adjusted

Active smoking at any time during pregnancy was associated with a decrease in the relative abundance of *Akkermansia*. Sustained maternal smoking from the first to the third trimester of pregnancy was associated with an increased relative abundance of *Dorea*. Exposures to tobacco (151 children) were adjusted by maternal education, maternal BMI, and having siblings at birth (previously selected by the *envfit* function). Exposure to mercury during pregnancy (148 children) was adjusted by original data of fish intake at 32 weeks of pregnancy, maternal education, and having siblings at birth. Exposure to mercury at 4 years (151 children) was adjusted by fish intake at 4 years, maternal education, and having siblings at birth.

qByGenus, *q*-value obtained from the raw MaAsLin2 output table and calculated with the BH correction. MaAsLin2, multivariable association model.

## 4 Discussion

In this study, we explored the association of childhood or perinatal exposure to tobacco and mercury with children’s gut microbial community diversity measures and taxa. We considered a large range of important co-factors such as sociodemographic, clinical, dietary, and environmental factors of the fecal microbiome in a well-studied cohort of healthy children. Exposures to tobacco smoke and mercury are frequent in this population and have been well characterized throughout pregnancy and childhood. A potential persistent influence of prenatal exposure to tobacco smoke could be established on the abundance of specific taxa of the gut microbiota during childhood.

Most previous studies on the early-life determinants of the children’s gut microbiota have focused on their influence from birth to the age of 3 years, when the microbial population is changing. By contrast, we studied the microbiota of older children, at 7 years old, when its composition is more stable. Previous studies had investigated the influence of breastfeeding, antibiotics, or diet on child microbiota but we added the influence of two common pollutant exposures at birth and during childhood. We determined the community structure of the gut microbiota in this 151 children population and investigated associations of accurately measured main pollutant exposures with the gut microbiota.

### 4.1 Distribution of tobacco smoke and mercury exposures

In the studied population, a high level exposure to tobacco smoke *in utero* and during childhood was found.

Median quantification of mercury from cord blood was equivalent to 4.5 μg/L in maternal blood, which was above the maximum mean value reported through the HELIX cohort (0.7–3.9 μg/L) (Aurrekoetxea et al., 2013). In this study, Mercury levels in cord blood samples were above 5.8 μg/L for a high proportion (65%) of children as found in communities with high fish intake such as Japan, Hong Kong, Taiwan, and Polynesia (geometric median 9.8, 8.8, 9.2, and 10.5 μg/L, respectively) (Ruggieri et al., 2017). Higher blood cord mercury levels have only been found in Canada or Greenland (geometric median 18.5 and 25.3 μg/L, respectively). Hair mercury levels above 1 μg/g were detected for a high proportion of children (almost 40%). These high values could be related to the place of residence of the studied population, where high fish and other aquatic food intake was the rule. Exposure to both tobacco smoke and upper quartile level of mercury was found in 12.1% of children at birth and 3.45% of 4-year-old children. For example, in comparison, only 8% of the children included in the DEMOCOPHES cohort implemented in 17 European countries exceeded this health-based value (Esteban et al., 2015).

### 4.2 Impact of tobacco smoke exposure and mercury on gut microbiota composition

The gut microbiota in healthy children of 7 years old showed a predominance of *Bacteroides*, *Akkermansia*, *Alistipes*, and *Faecalibacterium*, as described previously (Xiong et al., 2022). Based on the results of a multideterminant model, active smoking during pregnancy was the most associated determinant with the children’s gut microbiome β-diversity (Aitchison distance). Among all the other possible determinants included in the study, having siblings at birth was significantly associated with the β-diversity. Nevertheless, no significant associations between smoking variables with the β-diversity were observed in the univariate adjusted model.

We tried to determine the significant factors driving differences in the individual gut microbiota at the genus level. Maternal smoking during pregnancy was related to a decreased relative abundance of beneficial genus (*Akkermansia*) and an increased relative abundance of potentially pathogenic genus (*Dorea*), suggesting the importance of the environmental determinants during pregnancy, as a potential window of vulnerability. We did not find any other persistent effects at the genus level for tobacco exposure during childhood.

This study assessed cord blood and hair mercury levels as markers for mercury absorption and, consequently, mercury exposure. These levels elevate in correlation with the mother’s and child’s fish consumption, respectively, as well as the mercury content in the consumed fish species. Therefore, fish intake was regarded as a confounding factor in the analysis. It has been well established that intestinal microbiota can influence methylmercury intestinal absorption and the induction of intestinal dysbiosis (Pinto et al., 2020). On the other hand, methylmercury could have adverse effects on gut bacteria and accelerated accumulation of mercury in organs due to disruption of gut microbiota. Nevertheless, a sustained influence of early life mercury exposure could not be demonstrated in this study at the genus level. As it was suggested that mercury could decrease the growth of *Lactobacillus* in different proportions depending on the species (Seki et al., 2021), an additional study at the species level using a shotgun strategy could be relevant. In addition, as changes in the overall composition of the microbiome have been described for mercury exposure during early gestation, but not at late gestation (Rothenberg et al., 2016), it would be interesting to study other indicators of early exposure to mercury. Finally, the exposure to mercury at the most vulnerable stage of growth, during brain development, could lead to neurotoxicity in the unborn infant by a mechanism independent of permanent changes in microbiota. Higher childhood or perinatal blood mercury has been related to higher relative abundance of potential pathogens (e.g., *Flavonifractor plautii*), beneficial species (e.g., *Bifidobacterium longum*, *Faecalibacterium prausnitzii*), and both potentially pathogenic and beneficial species (e.g., *Bacteriodes vulgatus*, *Eubacterium rectale*), but these results were not found in our study (Shen et al., 2022).

Cigarette smoking has been consistently linked to alterations in the gut microbiome among adults (Cicchinelli et al., 2023). The impact of maternal smoking during gestation on the prenatal and perinatal gut environment is also suspected. These alterations may be influenced by shifts in the mother’s own microbiome or variations in the *in utero* chemical environment. Consequently, such changes could potentially reshape the trajectory of the microbiome development of offspring, rendering them more susceptible to inflammation. For example, changes in the abundances of several genera of the child microbiome of the finger, nose, mouth, and ear canal have been reported after exposure to third-hand smoke. These genera are also affected by active smoking and second-hand smoke (e.g., *Corynebacterium, Staphylococcus, Streptococcus*) (Kelley et al., 2021). Similarly, the abundance of *Serratia, Moraxella, Haemophilus*, and *Staphylococcus aureus* in tracheal aspirate changed after tobacco exposure (Leroue et al., 2023). The longitudinal effects of environmental tobacco smoke exposure on gut microbiota of young children have been previously reported. Tobacco was correlated with *Megasphaera* abundance. Breastfeeding was correlated with *Lactobacillus* abundance and negatively correlated with *Clostridium_sensu_stricto*_1 abundance (Xie et al., 2021).

Previous microbiome studies have also shown the inverse relationship between the abundance of *Akkermansia muciniphila*, a mucus-layer-degrading bacterium, and diseases such as inflammatory bowel disease, obesity, and diabetes. Its potential as immunomodulatory probiotic for autoimmune and chronic inflammatory diseases has been explored in experimental models. This species seems to slow down the development and progression of diabetes, obesity, and IBD in mice (Rodrigues et al., 2022). On the other side, *Phascolarctobacterium* has been previously associated with colorectal cancer (Bucher-Johannessen et al., 2023). This suggests a long term effect of tobacco early-life exposure on gut microbiome of children.

It was previously reported that at 1, 3, and 6 months of age, *Staphylococcus, Ruminococcus, Akkermansia, Lachnospiraceae*, and *Bacteroides* were more dominant in the gut microbiota of infants and neonates exposed to tobacco smoke (McLean et al., 2019). Further studies would be necessary to explore the effect of smoking on specific species or strains of these genera by using shotgun metagenomic analysis, allowing a more detailed view of the microbiome and its function.

Several strengths of our study can be highlighted. First, highly sensitive biomonitoring methods were used for tobacco and mercury assessment, combined with a longitudinal design (repeated measurements). In addition, this population has particularly high exposure to tobacco and mercury, which makes it more likely to detect potential effects, even for a relatively small size in the omics field. The INMA children are a well-characterized, general population, where the width of parameters was measured, which is relevant for gut microbial diversity description. It was particularly important to be able to compare the effect of pollutants of interest, a novelty in the field of microbiome determinants, with established factors, such as diet and clinical factors.

The main limitations of this study are that samples were stored at 4°C for more than 1.5 h, and that the information of antibiotic intake in the previous month was not recorded. Considering that strong clustering by individual is expected in fecal samples because inter-individual variability could be greater than the variability associated with storage time (Holzhausen et al., 2021) and based on the results of alpha diversity of this dataset (data not shown), we considered that our samples were adequate for an exploratory analysis. Besides, unknown exposures that could be influencing the microbial composition as other chemicals, endocrine disruptors, heavy metals, air pollution, pesticides, food additives, air pollution in the place where children were raised were not included.

The causal link between the determinants and gut microbiota composition could not be inferred from this association study, as associations might be confounded by other factors. Exposure data collected at the age of 4 years might have changed at microbiota assessment age.

## 5 Conclusion

Our findings suggest that early life exposome determinants, in particular, tobacco exposure during pregnancy, could have a long-term sustainable effect on the gut microbiota of children, at least at the same level as the diet, an established determinant of the gut microbiota diversity. Assessing the role of tobacco and mercury exposure on the microbiota of children, considering multiple environmental exposures, should be further investigated.

Clinical follow-up of individuals of this birth cohort, and increasing the sample size by combining other cohorts with similar protocols, would contribute to determining if these changes would impact the health of children. The influence of exposures on the gut microbiota of children seems to be a promising topic to focus on.

## Data availability statement

The datasets presented in this study can be found in online repositories. The names of the repository/repositories and accession number(s) can be found below: https://www.ebi.ac.uk/metagenomics/, PRJEB69271.

## Ethics statement

The studies involving humans were approved by the Ethics Committee of the Institut Municipal d’Investigacio Medica (IMIM), Barcelona, Spain. The studies were conducted in accordance with the local legislation and institutional requirements. Written informed consent for participation in this study was provided by the participants’ legal guardians/next of kin.

## Author contributions

SP: Formal analysis, Software, Writing – original draft, Writing – review & editing. GD’A: Formal analysis, Investigation, Writing – review & editing. ML: Conceptualization, Formal analysis, Investigation, Methodology, Writing – review & editing. SF-B: Investigation, Writing – review & editing. M-JL-E: Investigation, Writing – review & editing. SL: Investigation, Methodology, Writing – review & editing. BR: Writing – review & editing. MB: Conceptualization, Investigation, Methodology, Supervision, Writing – review & editing, Funding acquisition. MF: Conceptualization, Investigation, Methodology, Supervision, Writing – review & editing, Funding acquisition. MV: Investigation, Writing – review & editing. LM: Conceptualization, Data curation, Formal analysis, Funding acquisition, Investigation, Methodology, Supervision, Validation, Writing – review & editing.

## Footnotes

^1^ http://www.R-project.org/
