# Supplementary material for: Influence of perinatal and childhood exposure to tobacco and mercury in children’s gut microbiota
Source: Front Microbiol. 2024 Jan 5;14:1258988. doi: 10.3389/fmicb.2023.1258988 (PMC10799562; doi:10.3389/fmicb.2023.1258988)
Supplement: Supplementary file 1 [file Data_Sheet_1.pdf]

Supplementary Material

Influence of perinatal and childhood exposure to tobacco and mercury in children´s gut microbiota

Sonia Pérez-Castro*, Giuseppe D'Auria, Maria Llambrich, Sílvia Fernández-Barrés, Maria-Jose Lopez-Espinosa, Sabrina Llop, Benito Regueiro, Mariona Bustamante, M. Pilar Francino, Martine Vrijheid, Léa Maitre*

*** Correspondence:** Corresponding Authors: lea.maitre@isglobal.org and sonia.maria.perez.castro@sergas.es

1 Supplementary Tables

Supplementary Table S1. Description of the variables investigated in this study and their categories. Labels correspond to the names in tables and figures in the manuscript. FFQ: Food frequency questionnaire. Cigarettes/day (c/d)

Period	Type	Categories	Variable description	Label
Pregnancy	Clinical records		Mother BMI pre-pregnancy in kg/m^2	Maternal BMI
Pregnancy	Pollutants		Mercury - imputed birth cord blood	Mercury at birth
Pregnancy	Pollutants		Mean number of cigarettes/day during pregnancy	Cig pregnancy
Pregnancy	Pollutants	No, yes	Maternal active smoking at any time during pregnancy	Active smoke pregnancy
Pregnancy	Pollutants	No, yes	Sustained maternal smoking (T1 and T3)	Sustained smoke pregnancy
Pregnancy	Pollutants	0: no exposure<br>1: only passive<br>2: non-sustained<br>3: low dose (≤9 c/d)<br>4: high_dose (>9 c/d)<br>Grouped as:	Maternal smoking dose-duration	Maternal smoking dose-duration

Period	Type	Categories	Variable description	Label
		0: no exposure<br>1: only passive<br>2: non-sustained/ low dose (≤9 c/d)/ high_dose (>9 c/d)		
Pregnancy	Pollutants		Detectable cotinine levels (≥4 ng/mL)	SHSUrinaryCotinine_Preg
Pregnancy	Pollutants		Maternal urinary cotinine levels	Maternal cotinine levels
Birth	Clinical records	No, yes	Caesarean delivery	Caesarean delivery
Birth	Demographics		Siblings at birth	Siblings at birth
Infancy	Clinical records		sum_DTPa_Vpi_Hib_MCC	Vaccination group
Infancy	Clinical records		XRP	Vaccination XRP
Infancy	Clinical records		HB	Vaccination HB
Infancy	Demographics	Boys, girls	Sex	Sex
Infancy	Demographics	Others, WhiteEuropeans	Ethnic origin	Ethnic origin
Infancy	Diet	>16 weeks<br>0-16 weeks<br>0 weeks	Predominant breastfeeding	Breastfeeding
Infancy	Genetic		Principal component GWAS 1	Genetic1
Infancy	Genetic		Principal component GWAS 2	Genetic2
Infancy	Genetic		Principal component GWAS 3	Genetic3
Infancy	Genetic		Principal component GWAS 4	Genetic4
Infancy	Genetic		Principal component	Genetic5

Period	Type	Categories	Variable description	Label
			GWAS 5	
Infancy	Genetic	Se_, sese	FUT2 (rs601338)	FUT2 (rs601338)
Infancy	Quality of living and social environment	No, yes	Day care attendance at age 2y	Day care
Infancy	Quality of living and social environment	No, yes	Pet ownership at age 14 mo	Pets at 14 months
Infancy	Quality of living and social environment	High, Low, Medium. Grouped as: High, Low-Medium	Maternal education	Maternal education
4yrs	Diet		Vegetables intake in grams/day, FFQ 4 yrs	Vegetables_4yrs
4yrs	Diet		Fruits intake in grams/day, FFQ 4 yrs	Fruits_4yrs
4yrs	Diet		Nuts intake in grams/day, FFQ 4 yrs	Nuts_4yrs
4yrs	Diet		Legumes intake in grams/day, FFQ 4 yrs	Legumes_4yrs
4yrs	Diet		Sweets and sugar intake in grams/day, FFQ 4 yrs	Sweets ans sugars_4yrs
4yrs	Diet		Ready-made food intake in grams/day, FFQ 4 yrs	Ready-made food_4yrs
4yrs	Diet		Dairy products intake in grams/day, FFQ 4 yrs	Dairy_4yrs
4yrs	Diet		Processed meat intake in grams/day, FFQ 4 yrs	Processed meat_4yrs
4yrs	Diet		Meat intake in grams/day, FFQ 4 yrs	Meat_4yrs
4yrs	Diet		Fatty fish intake in grams/day, FFQ 4 yrs	Fatty fish_4yrs

Period	Type	Categories	Variable description	Label
4yrs	Diet		Seafood intake in grams/day, FFQ 4 yrs	Seafood_4yrs
4yrs	Diet		Fish intake in grams/day, FFQ 4 yrs	Fish_4yrs
4yrs	Diet	1: ≥3/week<br>2: 2-3/week<br>3: 1/week<br>4: 1-3/month<br>5: never<br>9: na/nk	How often does your child eat or snack in FAST FOOD restaurants?	Fast food_4yrs
4yrs	Diet	0: no<br>1: yes	During the past year, has your child taken vitamin or mineral supplements?	Vitamin_sup_4yrs
4yrs	Diet		Vit 25OH D3 (ng/ml)	VitD_4yrs
4yrs	Pollutants		Mercury - child hair 4 yrs	Mercury 4yrs
4yrs	Pollutants	No, yes	Secondhand smoke exposure at home	Secondhandsmoke_home_4yrs
4yrs	Pollutants	No, yes	Global secondhand smoke exposure	Secondhandsmoke_all_4yrs
4yrs	Pollutants	No, yes	Detectable cotinine levels (>=4 ng/mL)	SHSUrinaryCotinine_4yrs
4yrs	Pollutants		Urinary cotinine levels	Cotinine levels_4yrs
7yrs	Clinical records	Underweight/normal (normal_weigh), overweight/obese (Ovg-Obese)	BMI status	BMI status
7yrs	Clinical records	No, yes	Any medicines for asthma/breathing difficulties in last 12 mo?	Asthma
7yrs	Clinical records	No, yes	Medicines, tablets, nasal sprays... against nasal allergy/hay fever in past 12 mo	Hayfever_allergies

Period	Type	Categories	Variable description	Label
7yrs	Clinical records	No, yes	Has the child been suffering from infectious disease in the past 3 years? (chickenpox, rubella, scarletfever, exanthematic)	Infection
7yrs	Demographics		Child age	Child age
7yrs	Demographics	No, yes	Has the child any siblings (older or younger)?	Siblings_7yrs
7yrs	Diet		Dairy products intake in grams/day, FFQ 7 yrs	Dairy_7yrs
7yrs	Diet		Processed meat intake in grams/day, FFQ 7 yrs	Processed meat_7yrs
7yrs	Diet		Meat intake in grams/day, FFQ 7 yrs	Meat_7yrs
7yrs	Diet		Fatty fish intake in grams/day, FFQ 7 yrs	Fatty fish_7yrs
7yrs	Diet		Seafood intake in grams/day, FFQ 7 yrs	Seafood_7yrs
7yrs	Diet		Fish intake in grams/day, FFQ 7 yrs	Fish_7yrs
7yrs	Diet		Vegetables intake in grams/day, FFQ 7 yrs	Vegetables_7yrs
7yrs	Diet		Fruits intake in grams/day, FFQ 7 yrs	Fruits_7yrs
7yrs	Diet		Legumes intake in grams/day, FFQ 7 yrs	Legumes_7yrs
7yrs	Diet		Nuts intake in grams/day, FFQ 7 yrs	Nuts_7yrs
7yrs	Diet		Sweets and sugar intake in grams/day, FFQ 7 yrs	Sweets and sugars_7yrs
7yrs	Diet		Ready-made food intake in grams/day, FFQ 7 yrs	Ready-made food_7yrs

Period	Type	Categories	Variable description	Label
7yrs	Diet	0 False<br>1 True	Goes >1/ week to a fast food restaurant (hamburger)	Fast food_7yrs
7yrs	Diet	High, Poor-Medium	KIDMED	Healthy diet_7yrs
7yrs	Pollutants	No, yes	Secondhand smoke exposure at home	Secondhandsmoke_home
7yrs	Pollutants	No, yes	Global secondhand smoke exposure	Secondhandsmoke_all
7yrs	Quality of living and social environment		How many hours on average per day sleep including naps?	Sleep
7yrs	Quality of living and social environment	1: Sits down (taking, reading a book or playing sedentary games)<br>2: Stands around or walk around<br>3: Runs and play a little bit<br>4: Runs and play quite a bit<br>5: Runs and play hard most of the time<br>Grouped as:<br>1: Stands, walk or runs and play a little bit<br>2: Runs and play hard most of the time<br>3: Runs and play quite a bit	Your child usually...	Physical activity

Supplementary Table S2. Descriptive of the characteristics of the studied population, classified in 6 categories (diet, demographics, quality of living, clinical records, genetics and exposure to 2 pollutants -tobacco and mercury-), and in 3 periods (from pregnancy to infancy, at 4 years of age and at 7 years of age). Animal protein foods, dairy products, fruits and vegetables, high-fiber foods and sweet products are expressed as g/day. Mean of cigarettes/day during pregnancy 0.8123. Median [25%;75%] were indicated for continuous variables. Frequency (%) were indicated for categorical variables.

PERIOD	TYPE	VARIABLE DESCRIPTION	n (%); Median [25%,75%]	N
Pregnancy	Clinical records	Maternal BMI	22.72 [21.14,25.48]	151
	Diet	Fish Intake at 32 weeks pregnancy	426.69 [303.55,575.26]	148
	Pollutants	Active_smoke_pregnancy	36 (24%)	148
		cigarettes/day during pregnancy	0.00 [0.00,0.00]	148
		Maternal cotinine levels (µg/g)	6.04 [3.32,14.10]	148
		Maternal_smoking_dose_duration		148
		no exposure	93 (63%)	
		only passive	39 (26%)	
		non-sustained/low/high	16 (11%)	
		Mercury at birth (µg/L)	7.70 [4.80,14.00]	120
		SHSUrinaryCotinine_Preg		148
		not exposed	79 (53%)	
		exposed	69 (47%)	
		Sustained_smoke_pregnancy	14 (9.5%)	148
Birth	Clinical records	Caesarean_delivery	20 (13%)	149

	Demographics	Siblings_at_birth	64 (43%)	149
Infancy	Clinical records	Vaccination XRP	3 [2,3]	151
	Clinical records	Vaccination HB	3 [3,3]	151
	Clinical records	Vaccination group	16 [15,16]	149
	Diet	Breastfeeding		151
		0 weeks	29 (19%)	
		0-16 weeks	45 (30%)	
		>16 weeks	77 (51%)	
	Quality of living and social environment	Day_care	130 (88%)	147
		Pets_at_14_months	46 (30%)	151
	Demographics	Ethnic_origin		150
		WhiteEuropeans	140 (93%)	
		Others	10 (6.7%)	
		Sex		151
		Boys	78 (52%)	
		Girls	73 (48%)	
	Genetic	FUT2		107
		(GG+GA)	92 (86%)	
		sese(AA)	15 (14%)	

		Genetic1	0.02 [0.01,0.02]	107
		Genetic2	0.00 [-0.02,0.02]	107
		Genetic3	0.00 [-0.02,0.01]	107
		Genetic4	0.00 [-0.02,0.02]	107
		Genetic5	0.00 [-0.02,0.02]	107
	Quality of living and social environment	Maternal_education		150
		High	64 (43%)	
		Low_Med	86 (57%)	
4 years	Diet	Dairy_4yrs	463.86 [320.96,599.32]	150
		Fast_food_4yrs		149
		1/week	9 (6.0%)	
		1-3/month	103 (69%)	
		never	37 (25%)	
		Fatty fish_4yrs	7.60 [3.58,10.15]	150
		Fish_4yrs	35.47 [26.09,46.32]	150
		Fruits_4yrs	136.66 [94.83,199.47]	150
		Legumes_4yrs	12.60 [8.58,23.58]	150
		Meat_4yrs	88.02 [71.69,107.82]	150
		Nuts_4yrs	1.34 [0.00,2.86]	150

		Processed meat_4yrs	32.69 [24.11,40.64]	150
		Ready-made food_4yrs	86.93 [76.23,102.14]	150
		Seafood_4yrs	8.58 [4.73,12.60]	150
		Sweets and sugars_4yrs	44.88 [32.18,59.47]	150
		Vegetables_4yrs	57.47 [37.45,84.17]	150
		Vitamin_sup_4yrs	38 / 149 (26%)	149
		VitD_4yrs	36.78 [24.74,46.83]	97
	Pollutants	Cotinine levels_4yrs (µg/g)	5.51 [3.59,10.79]	145
		Mercury 4 years (µg/g)	0.82 [0.50,1.45]	138
		Secondhandsmoke_all_4yrs	81 (58%)	140
		Secondhandsmoke_home_4yrs	43 (32%)	134
		SHSUrinaryCotinine_4yrs		145
		not exposed	101 (70%)	
		exposed	44 (30%)	
7 years	Clinical records	Asthma	21 (14%)	148
		BMI_status		147
		normal_weight	104 (71%)	
		Ovg-Obese	43 (29%)	
		Hayfever_allergies	16 (11%)	148

		Infection		148
		No	131 (89%)	
		Yes	17 (11%)	
	Demographics	Child age (years)	6.75 [6.38,7.06]	147
		Siblings_7yrs	67 (44%)	151
	Diet	Dairy_7yrs	509.65 [309.83,569.31]	148
		Fast_food_7yrs		147
		false	136 (93%)	
		true	11 (7.5%)	
		Fatty fish_7yrs	9.38 [4.02,20.82]	148
		Fish_7yrs	47.19 [34.32,61.29]	148
		Fruits_7yrs	153.68 [100.00,250.90]	148
		Healthy_diet_7yrs		147
		Poor-Medium	76 (52%)	
		High	71 (48%)	
		Legumes_7yrs	1.34 [0.00,2.86]	148
		Meat_7yrs	103.60 [90.81,113.68]	148
		Nuts_7yrs	51.48 [17.16,51.48]	148
		Processed meat_7yrs	30.75 [22.17,40.76]	148

		Ready-made food_7yrs	32.80 [23.86,40.76]	148
		Seafood_7yrs	4.29 [2.01,10.59]	148
		Sweets and sugars_7yrs	42.10 [27.38,57.10]	148
		Vegetables_7yrs	94.38 [58.18,151.48]	148
	Pollutants	Secondhandsmoke_all	42 (29%)	147
		Secondhandsmoke_home	30 (20%)	147
	Quality of living and social environment	Physical_activity		145
		Stands, walk or runs and play a little bit	54 (37%)	
		Runs and play hard most of the time	33 (23%)	
		Runs and play quite a bit	58 (40%)	
		Sleep (hours)	10.00 [9.50,10.50]	148

Supplementary Table S3. β-Diversity multivariate analysis results (envfit for 151 children). Mean and standard deviation of R^2 and mean of *p*-value obtained for 20 imputations are shown. The number of significant imputations are indicated in the column "sign imput". This analysis was performed for wUnifrac, Bray-Curtis and Aitchison distances.

	wUnifrac				Bray-Curtis				Aitchison			
	mean R2	sdR2	Mean Pval	Sign imput	Mean R2	sdR2	Mean Pval	Sign imput	Mean R2	sdR2	Mean Pval	Sign imput
Active_smoke_pregnancy	0.0099	0.0009	0.2181	0	0.0149	0.0062	0.1327	3	0.0381	0.0064	0.0045	20
Asthma	0.0071	0.0008	0.3235	0	0.0129	0.0020	0.1424	0	0.0105	0.0012	0.2021	0
BMI_status	0.0151	0.0013	0.1046	0	0.0107	0.0009	0.1933	0	0.0087	0.0007	0.2627	0
Breastfeeding	0.0094	0.0000	0.5545	0	0.0050	0.0002	0.7944	0	0.0039	0.0000	0.8427	0
Caesarean_delivery	0.0082	0.0028	0.3023	0	0.0019	0.0009	0.7194	0	0.0013	0.0003	0.7843	0
Child_age	0.0082	0.0011	0.5176	0	0.0039	0.0010	0.7147	0	0.0005	0.0002	0.9191	0
Cig_pregnancy	0.0123	0.0019	0.4287	0	0.0218	0.0140	0.2632	5	0.0203	0.0074	0.2349	3
Cotinine_levels_4yrs	0.0409	0.0051	0.0500	9	0.0062	0.0016	0.5967	0	0.0234	0.0024	0.1678	0
Dairy_4yrs	0.0212	0.0007	0.1944	0	0.0041	0.0005	0.7041	0	0.0031	0.0008	0.7579	0
Dairy_7yrs	0.0421	0.0031	0.0419	13	0.0256	0.0024	0.1412	0	0.0132	0.0005	0.3575	0
Day_care	0.0008	0.0008	0.8526	0	0.0029	0.0006	0.6133	0	0.0013	0.0001	0.7934	0
Ethnic_origin	0.0042	0.0021	0.5265	0	0.0038	0.0001	0.5147	0	0.0057	0.0002	0.4066	0
Fast_food_4yrs	0.0132	0.0004	0.3860	0	0.0079	0.0011	0.6271	0	0.0048	0.0011	0.7991	0
Fast_food_7yrs	0.0073	0.0008	0.3130	0	0.0055	0.0018	0.4145	0	0.0135	0.0019	0.1364	0
Fatty_fish_4yrs	0.0261	0.0007	0.1329	0	0.0033	0.0005	0.7478	0	0.0033	0.0003	0.7444	0
Fatty_fish_7yrs	0.0069	0.0011	0.5720	0	0.0122	0.0011	0.3891	0	0.0164	0.0018	0.2881	0
Fish_4yrs	0.0170	0.0006	0.2676	0	0.0047	0.0009	0.6750	0	0.0107	0.0010	0.4325	0
Fish_7yrs	0.0100	0.0007	0.4613	0	0.0172	0.0019	0.2671	0	0.0348	0.0009	0.0717	1
Fruits_4yrs	0.0013	0.0003	0.8585	0	0.0144	0.0024	0.3302	0	0.0107	0.0021	0.4402	0
Fruits_7yrs	0.0151	0.0015	0.3105	0	0.0242	0.0016	0.1567	0	0.0204	0.0008	0.2128	0
Hayfever_allergies	0.0056	0.0007	0.4085	0	0.0060	0.0010	0.3829	0	0.0004	0.0002	0.8915	0
Healthy_diet_7yrs	0.0061	0.0010	0.3729	0	0.0041	0.0006	0.5261	0	0.0034	0.0006	0.5761	0
Infection	0.0027	0.0008	0.6347	0	0.0099	0.0025	0.2281	0	0.0082	0.0017	0.2882	0
Legumes_4yrs	0.0015	0.0005	0.7644	0	0.0063	0.0008	0.3204	0	0.0131	0.0017	0.1357	0
Legumes_7yrs	0.0027	0.0006	0.6216	0	0.0012	0.0003	0.8063	0	0.0124	0.0006	0.1508	0
Maternal_BMI	0.0266	0.0000	0.1300	0	0.0094	0.0004	0.4482	0	0.0163	0.0000	0.2832	0
Maternal_cotinine_levels	0.0092	0.0030	0.4923	0	0.0014	0.0009	0.8591	0	0.0081	0.0013	0.5199	0
Maternal_education	0.0055	0.0002	0.4093	0	0.0029	0.0005	0.6229	0	0.0084	0.0001	0.2756	0
Maternal_smoking_dose_duration	0.0285	0.0026	0.0771	2	0.0254	0.0108	0.1551	4	0.0257	0.0064	0.1219	4
Meat_4yrs	0.0072	0.0001	0.5626	0	0.0131	0.0008	0.3555	0	0.0281	0.0027	0.1217	0
Meat_7yrs	0.0195	0.0021	0.2291	0	0.0094	0.0027	0.4690	0	0.0111	0.0027	0.4312	0
Mercury_4yrs	0.0115	0.0032	0.4205	0	0.0146	0.0041	0.3372	0	0.0015	0.0014	0.8592	0
Mercury_at_birth	0.0257	0.0101	0.1752	2	0.0185	0.0058	0.2656	0	0.0157	0.0064	0.3334	0
Nuts_4yrs	0.0087	0.0005	0.2571	0	0.0115	0.0005	0.1714	0	0.0188	0.0013	0.0583	4
Nuts_7yrs	0.0184	0.0020	0.0662	3	0.0119	0.0014	0.1627	0	0.0092	0.0008	0.2393	0
Pets_at_14_months	0.0140	0.0000	0.1217	0	0.0038	0.0003	0.5455	0	0.0061	0.0000	0.3866	0
Physical_activity	0.0013	0.0006	0.9315	0	0.0104	0.0033	0.5351	0	0.0177	0.0058	0.2942	0
Processed_meat_4yrs	0.0068	0.0002	0.5672	0	0.0011	0.0003	0.8712	0	0.0028	0.0004	0.7759	0
Processed_meat_7yrs	0.0216	0.0018	0.1904	0	0.0341	0.0025	0.0704	0	0.0214	0.0019	0.1986	0
Ready_made_food_4yrs	0.0232	0.0008	0.1711	0	0.0236	0.0015	0.1635	0	0.0166	0.0015	0.2787	0

	wUnifrac				Bray-Curtis				Aitchison			
	mean R2	sdR2	Mean Pval	Sign imput	Mean R2	sdR2	Mean Pval	Sign imput	Mean R2	sdR2	Mean Pval	Sign imput
Ready_made_food_7yrs	0.0075	0.0012	0.5477	0	0.0044	0.0010	0.6837	0	0.0048	0.0014	0.6709	0
Seafood_4yrs	0.0327	0.0017	0.0850	0	0.0013	0.0004	0.8669	0	0.0138	0.0007	0.3363	0
Seafood_7yrs	0.0068	0.0012	0.5759	0	0.0206	0.0025	0.2086	0	0.0257	0.0020	0.1411	0
Secondhandsmoke_all	0.0007	0.0007	0.8638	0	0.0013	0.0003	0.7923	0	0.0044	0.0008	0.4998	0
Secondhandsmoke_all_4yrs	0.0056	0.0019	0.4269	0	0.0005	0.0004	0.8899	0	0.0018	0.0009	0.7385	0
Secondhandsmoke_home	0.0017	0.0011	0.7356	0	0.0025	0.0005	0.6565	0	0.0048	0.0008	0.4758	0
Secondhandsmoke_home_4yrs	0.0071	0.0018	0.3372	0	0.0041	0.0014	0.5315	0	0.0072	0.0036	0.3645	0
Sex	0.0020	0.0000	0.6973	0	0.0121	0.0006	0.1545	0	0.0074	0.0000	0.3149	0
SHSUrinaryCotinine_4yrs	0.0078	0.0018	0.3064	0	0.0094	0.0025	0.2566	0	0.0231	0.0018	0.0322	18
SHSUrinaryCotinine_Preg	0.0039	0.0006	0.5335	0	0.0036	0.0004	0.5670	0	0.0087	0.0009	0.2586	0
Siblings_7yrs	0.0045	0.0000	0.4856	0	0.0006	0.0004	0.8685	0	0.0047	0.0000	0.4759	0
Siblings_at_birth	0.0113	0.0009	0.1768	0	0.0248	0.0026	0.0214	20	0.0340	0.0013	0.0065	20
Sleep	0.0287	0.0049	0.1185	1	0.0010	0.0003	0.8833	0	0.0040	0.0004	0.7055	0
Sustained_smoke_pregnancy	0.0082	0.0028	0.3024	0	0.0183	0.0126	0.1176	3	0.0139	0.0054	0.1476	3
Sweets_and_sugars_4yrs	0.0036	0.0002	0.7322	0	0.0312	0.0023	0.0935	0	0.0309	0.0005	0.0928	0
Sweets_and_sugars_7yrs	0.0018	0.0006	0.8315	0	0.0012	0.0007	0.8701	0	0.0018	0.0004	0.8374	0
Vaccination_group	0.0081	0.0011	0.5229	0	0.0005	0.0004	0.9146	0	0.0005	0.0004	0.9218	0
Vaccination_XRP	0.0175	0.0000	0.4781	0	0.0064	0.0003	0.8783	0	0.0075	0.0000	0.8554	0
Vegetables_4yrs	0.0185	0.0006	0.2429	0	0.0111	0.0014	0.4205	0	0.0213	0.0017	0.1930	0
Vegetables_7yrs	0.0096	0.0016	0.4695	0	0.0025	0.0007	0.7878	0	0.0048	0.0004	0.6695	0
Vitamin_sup_4yrs	0.0069	0.0005	0.3313	0	0.0202	0.0021	0.0466	11	0.0161	0.0024	0.0886	1
VitD_4yrs	0.0155	0.0134	0.4391	1	0.0060	0.0052	0.6541	0	0.0112	0.0086	0.5002	0

Supplementary Table S4. Sensitivity analysis considering pollutants exposure, non-genetic and genetic determinants was performed in a subset of 107 children. Results of the β-Diversity multivariate results (envfit). Mean and standard deviation of R2 and mean of p-value obtained for 20 imputations are shown. The number of significant imputations are indicated in the column "sign imput". This analysis was performed for Aitchison distance.

Variable	meanR2	sdR2	meanPval	sign imput
Active_smoke_pregnancy	0.0677	0.0009	0.0016	20
Asthma	0.0033	0.0007	0.6771	0
BMI_status	0.0028	0.0004	0.7070	0
Breastfeeding	0.0083	0.0000	0.7469	0
Caesarean_delivery	0.0054	0.0005	0.5465	0
Child_age	0.0084	0.0012	0.6151	0
Cig_pregnancy	0.0258	0.0005	0.2356	0
Cotinine_levels_4yrs	0.0598	0.0040	0.0398	14
Dairy_4yrs	0.0017	0.0002	0.8710	0
Dairy_7yrs	0.0111	0.0005	0.5365	0
Day_care	0.0057	0.0005	0.5223	0
Ethnic_origin	0.0100	0.0004	0.3387	0
Fast_food_4yrs	0.0190	0.0039	0.3944	0
Fast_food_7yrs	0.0100	0.0030	0.3584	0
Fatty_fish_4yrs	0.0149	0.0016	0.4349	0
Fatty_fish_7yrs	0.0170	0.0015	0.3929	0
Fish_4yrs	0.0201	0.0032	0.3375	0
Fish_7yrs	0.0489	0.0013	0.0706	0
Fruits_4yrs	0.0098	0.0029	0.5761	0
Fruits_7yrs	0.0051	0.0009	0.7269	0

FUT2_rs601338	0.0075	0.0000	0.4318	0
Hayfever_allergies	0.0009	0.0006	0.8696	0
Healthy_diet_7yrs	0.0007	0.0003	0.8875	0
Infection	0.0049	0.0014	0.5740	0
Legumes_4yrs	0.0249	0.0051	0.0767	8
Legumes_7yrs	0.0218	0.0011	0.0940	0
Maternal_BMI	0.0387	0.0000	0.1215	0
Maternal_cotinine_levels	0.0343	0.0034	0.1565	0
Maternal_education	0.0066	0.0003	0.4813	0
Maternal_smoking_dose_duration	0.0439	0.0017	0.0521	5
Meat_4yrs	0.0604	0.0067	0.0410	15
Meat_7yrs	0.0136	0.0010	0.4691	0
Mercury_4yrs	0.0039	0.0031	0.7892	0
Mercury_at_birth	0.0399	0.0175	0.1676	4
Nuts_4yrs	0.0214	0.0021	0.0993	0
Nuts_7yrs	0.0051	0.0009	0.5564	0
Pets_at_14_months	0.0069	0.0000	0.4618	0
Physical_activity	0.0104	0.0017	0.6675	0
Principal_component_GWAS_1	0.0205	0.0000	0.3216	0
Principal_component_GWAS_2	0.0272	0.0000	0.2317	0
Principal_component_GWAS_3	0.0011	0.0000	0.8943	0
Principal_component_GWAS_4	0.0207	0.0000	0.3291	0
Principal_component_GWAS_5	0.0450	0.0000	0.0863	0
Processed_meat_4yrs	0.0032	0.0015	0.8144	0

Processed_meat_7yrs	0.0086	0.0018	0.6186	0
Ready_made_food_4yrs	0.0037	0.0003	0.7852	0
Ready_made_food_7yrs	0.0003	0.0003	0.9383	0
Seafood_4yrs	0.0097	0.0022	0.5708	0
Seafood_7yrs	0.0209	0.0014	0.3132	0
Secondhandsmoke_all	0.0220	0.0010	0.0921	0
Secondhandsmoke_all_4yrs	0.0150	0.0038	0.2118	0
Secondhandsmoke_home	0.0125	0.0013	0.2559	0
Secondhandsmoke_home_4yrs	0.0287	0.0102	0.0726	9
Sex	0.0084	0.0000	0.3922	0
SHSUrinaryCotinine_4yrs	0.0223	0.0015	0.0944	0
SHSUrinaryCotinine_Preg	0.0041	0.0009	0.6219	0
Siblings_7yrs	0.0079	0.0000	0.4133	0
Siblings_at_birth	0.0455	0.0021	0.0081	20
Sleep	0.0045	0.0019	0.7631	0
Sustained_smoke_pregnancy	0.0233	0.0011	0.0853	0
Sweets_and_sugars_4yrs	0.0272	0.0002	0.2276	0
Sweets_and_sugars_7yrs	0.0228	0.0031	0.2961	0
Vaccination_group	0.0010	0.0009	0.9076	0
Vaccination_XRP	0.0145	0.0000	0.7746	0
Vegetables_4yrs	0.0366	0.0033	0.1403	0
Vegetables_7yrs	0.0403	0.0025	0.1140	0
Vitamin_sup_4yrs	0.0062	0.0022	0.5128	0
VitD_4yrs	0.0163	0.0127	0.4779	0

Supplementary Table S5. Univariate unadjusted and adjusted β-diversity association study (PERMANOVA). R^2 values, crude *p*-values and adjusted *q*-values obtained for the association of β-diversity measured by Aitchison distance matrices and the pollutant exposures (12 variables of exposure to tobacco, 2 variables of exposure to mercury). Exposures to tobacco (151 children) were adjusted by maternal education, maternal BMI and having siblings at birth (previously selected by the *envfit* function). Exposure to mercury during pregnancy (148 children) was adjusted by original data of fish intake at 32 weeks of pregnancy, maternal education and having siblings at birth. Exposure to mercury at 4 years (151 children) was adjusted by fish intake at 4 years, maternal education and having siblings at birth.

Abbreviation: imp, imputation number

**MERCURY UNADJUSTED**	**R2**	**p.val**	**imp**
Mercury_at_birth	0.0107	0.045	1
Mercury_at_birth1	0.0117	0.015	2
Mercury_at_birth2	0.0110	0.021	3
Mercury_at_birth3	0.0103	0.03	4
Mercury_at_birth4	0.0100	0.047	5
Mercury_at_birth5	0.0114	0.023	6
Mercury_at_birth6	0.0097	0.067	7
Mercury_at_birth7	0.0092	0.083	8
Mercury_at_birth8	0.0121	0.008	9
Mercury_at_birth9	0.0112	0.013	10
Mercury_at_birth10	0.0100	0.059	11
Mercury_at_birth11	0.0106	0.036	12
Mercury_at_birth12	0.0111	0.022	13
Mercury_at_birth13	0.0096	0.078	14
Mercury_at_birth14	0.0108	0.025	15
Mercury_at_birth15	0.0118	0.011	16
Mercury_at_birth16	0.0097	0.056	17
Mercury_at_birth17	0.0118	0.008	18
Mercury_at_birth18	0.0093	0.081	19
Mercury_at_birth19	0.0082	0.202	20
Mercury_4yrs	0.0075	0.271	1
Mercury_4yrs1	0.0094	0.082	2

Mercury_4yrs2	0.0083	0.151	3
Mercury_4yrs3	0.0079	0.227	4
Mercury_4yrs4	0.0089	0.124	5
Mercury_4yrs5	0.0077	0.256	6
Mercury_4yrs6	0.0081	0.181	7
Mercury_4yrs7	0.0089	0.106	8
Mercury_4yrs8	0.0090	0.105	9
Mercury_4yrs9	0.0083	0.184	10
Mercury_4yrs10	0.0104	0.037	11
Mercury_4yrs11	0.0101	0.035	12
Mercury_4yrs12	0.0078	0.243	13
Mercury_4yrs13	0.0082	0.186	14
Mercury_4yrs14	0.0100	0.047	15
Mercury_4yrs15	0.0088	0.127	16
Mercury_4yrs16	0.0080	0.196	17
Mercury_4yrs17	0.0084	0.166	18
Mercury_4yrs18	0.0084	0.137	19
Mercury_4yrs19	0.0086	0.128	20
**MERCURY ADJUSTED**	**R2**	**p.val**	**imp**
Mercury_4yrs	0.0075	0.269	1
Mercury_4yrs1	0.0094	0.082	2
Mercury_4yrs10	0.0104	0.036	11
Mercury_4yrs11	0.0101	0.033	12
Mercury_4yrs12	0.0078	0.236	13
Mercury_4yrs13	0.0082	0.181	14
Mercury_4yrs14	0.0100	0.046	15
Mercury_4yrs15	0.0088	0.126	16
Mercury_4yrs16	0.0080	0.194	17

Mercury_4yrs17	0.0084	0.164	18
Mercury_4yrs18	0.0084	0.133	19
Mercury_4yrs19	0.0086	0.127	20
Mercury_4yrs2	0.0083	0.145	3
Mercury_4yrs3	0.0079	0.222	4
Mercury_4yrs4	0.0089	0.12	5
Mercury_4yrs5	0.0077	0.248	6
Mercury_4yrs6	0.0081	0.18	7
Mercury_4yrs7	0.0089	0.103	8
Mercury_4yrs8	0.0090	0.104	9
Mercury_4yrs9	0.0083	0.179	10
Mercury_at_birth	0.0107	0.042	1
Mercury_at_birth1	0.0117	0.015	2
Mercury_at_birth10	0.0100	0.056	11
Mercury_at_birth11	0.0106	0.035	12
Mercury_at_birth12	0.0111	0.022	13
Mercury_at_birth13	0.0096	0.077	14
Mercury_at_birth14	0.0108	0.024	15
Mercury_at_birth15	0.0118	0.011	16
Mercury_at_birth16	0.0097	0.056	17
Mercury_at_birth17	0.0118	0.008	18
Mercury_at_birth18	0.0093	0.08	19
Mercury_at_birth19	0.0082	0.201	20
Mercury_at_birth2	0.0110	0.02	3
Mercury_at_birth3	0.0103	0.029	4
Mercury_at_birth4	0.0100	0.047	5
Mercury_at_birth5	0.0114	0.021	6
Mercury_at_birth6	0.0097	0.064	7

Mercury_at_birth7	0.0092	0.08	8
Mercury_at_birth8	0.0121	0.008	9
Mercury_at_birth9	0.0112	0.013	10
**TOBACCO UNADJUSTED**	**R2**	**p.val**	**imp**
Active_smoke_pregnancy	0.0071	0.366	1
Active_smoke_pregnancy1	0.0055	0.745	2
Active_smoke_pregnancy10	0.0055	0.726	11
Active_smoke_pregnancy11	0.0071	0.348	12
Active_smoke_pregnancy12	0.0062	0.55	13
Active_smoke_pregnancy13	0.0062	0.572	14
Active_smoke_pregnancy14	0.0062	0.568	15
Active_smoke_pregnancy15	0.0054	0.728	16
Active_smoke_pregnancy16	0.0062	0.554	17
Active_smoke_pregnancy17	0.0061	0.602	18
Active_smoke_pregnancy18	0.0055	0.719	19
Active_smoke_pregnancy19	0.0062	0.549	20
Active_smoke_pregnancy2	0.0062	0.551	3
Active_smoke_pregnancy3	0.0071	0.355	4
Active_smoke_pregnancy4	0.0055	0.709	5
Active_smoke_pregnancy5	0.0062	0.529	6
Active_smoke_pregnancy6	0.0055	0.762	7
Active_smoke_pregnancy7	0.0055	0.709	8
Active_smoke_pregnancy8	0.0062	0.582	9
Active_smoke_pregnancy9	0.0055	0.709	10
Cig_pregnancy	0.0101	0.918	1
Cig_pregnancy1	0.0101	0.93	2
Cig_pregnancy10	0.0125	0.598	11
Cig_pregnancy11	0.0107	0.864	12

Cig_pregnancy12	0.0101	0.929	13
Cig_pregnancy13	0.0107	0.877	14
Cig_pregnancy14	0.0125	0.61	15
Cig_pregnancy15	0.0101	0.933	16
Cig_pregnancy16	0.0089	0.986	17
Cig_pregnancy17	0.0107	0.872	18
Cig_pregnancy18	0.0101	0.928	19
Cig_pregnancy19	0.0107	0.851	20
Cig_pregnancy2	0.0101	0.914	3
Cig_pregnancy3	0.0135	0.438	4
Cig_pregnancy4	0.0101	0.916	5
Cig_pregnancy5	0.0113	0.789	6
Cig_pregnancy6	0.0101	0.92	7
Cig_pregnancy7	0.0101	0.919	8
Cig_pregnancy8	0.0101	0.922	9
Cig_pregnancy9	0.0113	0.794	10
Cotinine_levels_4yrs	0.0075	0.279	1
Cotinine_levels_4yrs1	0.0070	0.365	2
Cotinine_levels_4yrs10	0.0072	0.327	11
Cotinine_levels_4yrs11	0.0074	0.309	12
Cotinine_levels_4yrs12	0.0069	0.375	13
Cotinine_levels_4yrs13	0.0077	0.247	14
Cotinine_levels_4yrs14	0.0073	0.306	15
Cotinine_levels_4yrs15	0.0075	0.26	16
Cotinine_levels_4yrs16	0.0065	0.486	17
Cotinine_levels_4yrs17	0.0066	0.478	18
Cotinine_levels_4yrs18	0.0067	0.426	19
Cotinine_levels_4yrs19	0.0070	0.337	20

Cotinine_levels_4yrs2	0.0073	0.311	3
Cotinine_levels_4yrs3	0.0075	0.274	4
Cotinine_levels_4yrs4	0.0068	0.434	5
Cotinine_levels_4yrs5	0.0065	0.477	6
Cotinine_levels_4yrs6	0.0076	0.254	7
Cotinine_levels_4yrs7	0.0075	0.265	8
Cotinine_levels_4yrs8	0.0074	0.319	9
Cotinine_levels_4yrs9	0.0069	0.409	10
Maternal_cotinine_levels	0.0046	0.897	1
Maternal_cotinine_levels1	0.0052	0.806	2
Maternal_cotinine_levels10	0.0056	0.705	11
Maternal_cotinine_levels11	0.0056	0.735	12
Maternal_cotinine_levels12	0.0055	0.738	13
Maternal_cotinine_levels13	0.0056	0.745	14
Maternal_cotinine_levels14	0.0055	0.752	15
Maternal_cotinine_levels15	0.0047	0.893	16
Maternal_cotinine_levels16	0.0046	0.922	17
Maternal_cotinine_levels17	0.0048	0.873	18
Maternal_cotinine_levels18	0.0048	0.874	19
Maternal_cotinine_levels19	0.0048	0.863	20
Maternal_cotinine_levels2	0.0046	0.906	3
Maternal_cotinine_levels3	0.0056	0.713	4
Maternal_cotinine_levels4	0.0047	0.904	5
Maternal_cotinine_levels5	0.0055	0.719	6
Maternal_cotinine_levels6	0.0046	0.898	7
Maternal_cotinine_levels7	0.0048	0.857	8
Maternal_cotinine_levels8	0.0047	0.895	9
Maternal_cotinine_levels9	0.0046	0.916	10

Maternal_smoking_dose_duration	0.0122	0.651	1
Maternal_smoking_dose_duration1	0.0120	0.712	2
Maternal_smoking_dose_duration10	0.0122	0.662	11
Maternal_smoking_dose_duration11	0.0129	0.517	12
Maternal_smoking_dose_duration12	0.0146	0.285	13
Maternal_smoking_dose_duration13	0.0113	0.774	14
Maternal_smoking_dose_duration14	0.0122	0.682	15
Maternal_smoking_dose_duration15	0.0123	0.62	16
Maternal_smoking_dose_duration16	0.0122	0.681	17
Maternal_smoking_dose_duration17	0.0110	0.828	18
Maternal_smoking_dose_duration18	0.0110	0.831	19
Maternal_smoking_dose_duration19	0.0122	0.675	20
Maternal_smoking_dose_duration2	0.0116	0.76	3
Maternal_smoking_dose_duration3	0.0122	0.663	4
Maternal_smoking_dose_duration4	0.0120	0.677	5
Maternal_smoking_dose_duration5	0.0129	0.505	6
Maternal_smoking_dose_duration6	0.0110	0.818	7
Maternal_smoking_dose_duration7	0.0110	0.832	8
Maternal_smoking_dose_duration8	0.0110	0.821	9
Maternal_smoking_dose_duration9	0.0110	0.837	10
Secondhandsmoke_all	0.0064	0.514	1
Secondhandsmoke_all_4yrs	0.0065	0.475	1
Secondhandsmoke_all_4yrs1	0.0065	0.48	2
Secondhandsmoke_all_4yrs10	0.0069	0.416	11
Secondhandsmoke_all_4yrs11	0.0070	0.379	12
Secondhandsmoke_all_4yrs12	0.0069	0.387	13
Secondhandsmoke_all_4yrs13	0.0069	0.401	14
Secondhandsmoke_all_4yrs14	0.0065	0.495	15

Secondhandsmoke_all_4yrs15	0.0065	0.484	16
Secondhandsmoke_all_4yrs16	0.0065	0.502	17
Secondhandsmoke_all_4yrs17	0.0069	0.377	18
Secondhandsmoke_all_4yrs18	0.0064	0.507	19
Secondhandsmoke_all_4yrs19	0.0063	0.539	20
Secondhandsmoke_all_4yrs2	0.0065	0.547	3
Secondhandsmoke_all_4yrs3	0.0072	0.344	4
Secondhandsmoke_all_4yrs4	0.0063	0.538	5
Secondhandsmoke_all_4yrs5	0.0067	0.458	6
Secondhandsmoke_all_4yrs6	0.0063	0.536	7
Secondhandsmoke_all_4yrs7	0.0070	0.379	8
Secondhandsmoke_all_4yrs8	0.0070	0.359	9
Secondhandsmoke_all_4yrs9	0.0070	0.379	10
Secondhandsmoke_all1	0.0060	0.634	2
Secondhandsmoke_all10	0.0064	0.499	11
Secondhandsmoke_all11	0.0070	0.367	12
Secondhandsmoke_all12	0.0066	0.43	13
Secondhandsmoke_all13	0.0070	0.379	14
Secondhandsmoke_all14	0.0063	0.528	15
Secondhandsmoke_all15	0.0066	0.476	16
Secondhandsmoke_all16	0.0060	0.627	17
Secondhandsmoke_all17	0.0064	0.513	18
Secondhandsmoke_all18	0.0070	0.379	19
Secondhandsmoke_all19	0.0069	0.426	20
Secondhandsmoke_all2	0.0066	0.453	3
Secondhandsmoke_all3	0.0069	0.404	4
Secondhandsmoke_all4	0.0063	0.556	5
Secondhandsmoke_all5	0.0066	0.461	6

Secondhandsmoke_all6	0.0066	0.437	7
Secondhandsmoke_all7	0.0066	0.475	8
Secondhandsmoke_all8	0.0069	0.364	9
Secondhandsmoke_all9	0.0066	0.472	10
Secondhandsmoke_home	0.0075	0.277	1
Secondhandsmoke_home_4yrs	0.0056	0.7	1
Secondhandsmoke_home_4yrs1	0.0063	0.494	2
Secondhandsmoke_home_4yrs10	0.0069	0.397	11
Secondhandsmoke_home_4yrs11	0.0062	0.533	12
Secondhandsmoke_home_4yrs12	0.0052	0.788	13
Secondhandsmoke_home_4yrs13	0.0060	0.619	14
Secondhandsmoke_home_4yrs14	0.0067	0.45	15
Secondhandsmoke_home_4yrs15	0.0065	0.488	16
Secondhandsmoke_home_4yrs16	0.0063	0.546	17
Secondhandsmoke_home_4yrs17	0.0058	0.658	18
Secondhandsmoke_home_4yrs18	0.0061	0.592	19
Secondhandsmoke_home_4yrs19	0.0062	0.562	20
Secondhandsmoke_home_4yrs2	0.0065	0.493	3
Secondhandsmoke_home_4yrs3	0.0065	0.493	4
Secondhandsmoke_home_4yrs4	0.0063	0.535	5
Secondhandsmoke_home_4yrs5	0.0060	0.632	6
Secondhandsmoke_home_4yrs6	0.0062	0.571	7
Secondhandsmoke_home_4yrs7	0.0064	0.548	8
Secondhandsmoke_home_4yrs8	0.0063	0.527	9
Secondhandsmoke_home_4yrs9	0.0067	0.46	10
Secondhandsmoke_home1	0.0075	0.262	2
Secondhandsmoke_home10	0.0079	0.233	11
Secondhandsmoke_home11	0.0077	0.241	12

Secondhandsmoke_home12	0.0075	0.287	13
Secondhandsmoke_home13	0.0069	0.427	14
Secondhandsmoke_home14	0.0077	0.232	15
Secondhandsmoke_home15	0.0075	0.265	16
Secondhandsmoke_home16	0.0069	0.405	17
Secondhandsmoke_home17	0.0069	0.403	18
Secondhandsmoke_home18	0.0073	0.302	19
Secondhandsmoke_home19	0.0077	0.247	20
Secondhandsmoke_home2	0.0075	0.276	3
Secondhandsmoke_home3	0.0077	0.25	4
Secondhandsmoke_home4	0.0075	0.271	5
Secondhandsmoke_home5	0.0077	0.246	6
Secondhandsmoke_home6	0.0077	0.244	7
Secondhandsmoke_home7	0.0073	0.322	8
Secondhandsmoke_home8	0.0071	0.36	9
Secondhandsmoke_home9	0.0073	0.324	10
SHSUrinaryCotinine_4yrs	0.0048	0.862	1
SHSUrinaryCotinine_4yrs1	0.0057	0.678	2
SHSUrinaryCotinine_4yrs10	0.0048	0.863	11
SHSUrinaryCotinine_4yrs11	0.0055	0.693	12
SHSUrinaryCotinine_4yrs12	0.0055	0.766	13
SHSUrinaryCotinine_4yrs13	0.0051	0.815	14
SHSUrinaryCotinine_4yrs14	0.0051	0.822	15
SHSUrinaryCotinine_4yrs15	0.0049	0.848	16
SHSUrinaryCotinine_4yrs16	0.0057	0.665	17
SHSUrinaryCotinine_4yrs17	0.0057	0.684	18
SHSUrinaryCotinine_4yrs18	0.0057	0.688	19
SHSUrinaryCotinine_4yrs19	0.0050	0.835	20

SHSUrinaryCotinine_4yrs2	0.0055	0.737	3
SHSUrinaryCotinine_4yrs3	0.0050	0.839	4
SHSUrinaryCotinine_4yrs4	0.0052	0.801	5
SHSUrinaryCotinine_4yrs5	0.0055	0.747	6
SHSUrinaryCotinine_4yrs6	0.0050	0.831	7
SHSUrinaryCotinine_4yrs7	0.0049	0.868	8
SHSUrinaryCotinine_4yrs8	0.0057	0.701	9
SHSUrinaryCotinine_4yrs9	0.0051	0.844	10
SHSUrinaryCotinine_Preg	0.0065	0.502	1
SHSUrinaryCotinine_Preg1	0.0076	0.237	2
SHSUrinaryCotinine_Preg10	0.0065	0.48	11
SHSUrinaryCotinine_Preg11	0.0069	0.396	12
SHSUrinaryCotinine_Preg12	0.0069	0.37	13
SHSUrinaryCotinine_Preg13	0.0065	0.492	14
SHSUrinaryCotinine_Preg14	0.0065	0.512	15
SHSUrinaryCotinine_Preg15	0.0072	0.336	16
SHSUrinaryCotinine_Preg16	0.0068	0.423	17
SHSUrinaryCotinine_Preg17	0.0065	0.452	18
SHSUrinaryCotinine_Preg18	0.0072	0.325	19
SHSUrinaryCotinine_Preg19	0.0065	0.495	20
SHSUrinaryCotinine_Preg2	0.0065	0.494	3
SHSUrinaryCotinine_Preg3	0.0065	0.493	4
SHSUrinaryCotinine_Preg4	0.0068	0.424	5
SHSUrinaryCotinine_Preg5	0.0069	0.389	6
SHSUrinaryCotinine_Preg6	0.0072	0.331	7
SHSUrinaryCotinine_Preg7	0.0065	0.492	8
SHSUrinaryCotinine_Preg8	0.0072	0.349	9
SHSUrinaryCotinine_Preg9	0.0078	0.229	10

Sustained_smoke_pregnancy	0.0050	0.845	1
Sustained_smoke_pregnancy1	0.0050	0.823	2
Sustained_smoke_pregnancy10	0.0068	0.406	11
Sustained_smoke_pregnancy11	0.0068	0.428	12
Sustained_smoke_pregnancy12	0.0068	0.418	13
Sustained_smoke_pregnancy13	0.0068	0.426	14
Sustained_smoke_pregnancy14	0.0068	0.425	15
Sustained_smoke_pregnancy15	0.0062	0.543	16
Sustained_smoke_pregnancy16	0.0068	0.443	17
Sustained_smoke_pregnancy17	0.0068	0.425	18
Sustained_smoke_pregnancy18	0.0046	0.929	19
Sustained_smoke_pregnancy19	0.0068	0.427	20
Sustained_smoke_pregnancy2	0.0068	0.433	3
Sustained_smoke_pregnancy3	0.0068	0.401	4
Sustained_smoke_pregnancy4	0.0050	0.835	5
Sustained_smoke_pregnancy5	0.0068	0.432	6
Sustained_smoke_pregnancy6	0.0069	0.384	7
Sustained_smoke_pregnancy7	0.0050	0.839	8
Sustained_smoke_pregnancy8	0.0069	0.392	9
Sustained_smoke_pregnancy9	0.0068	0.417	10
**TOBACCO ADJUSTED**	**R2**	**p.val**	**imp**
Active_smoke_pregnancy	0.0071	0.369	1
Active_smoke_pregnancy1	0.0055	0.741	2
Active_smoke_pregnancy10	0.0055	0.725	11
Active_smoke_pregnancy11	0.0071	0.343	12
Active_smoke_pregnancy12	0.0062	0.542	13
Active_smoke_pregnancy13	0.0062	0.567	14
Active_smoke_pregnancy14	0.0062	0.564	15

Active_smoke_pregnancy15	0.0054	0.727	16
Active_smoke_pregnancy16	0.0062	0.553	17
Active_smoke_pregnancy17	0.0061	0.6	18
Active_smoke_pregnancy18	0.0055	0.719	19
Active_smoke_pregnancy19	0.0062	0.546	20
Active_smoke_pregnancy2	0.0062	0.543	3
Active_smoke_pregnancy3	0.0071	0.355	4
Active_smoke_pregnancy4	0.0055	0.705	5
Active_smoke_pregnancy5	0.0062	0.525	6
Active_smoke_pregnancy6	0.0055	0.759	7
Active_smoke_pregnancy7	0.0055	0.707	8
Active_smoke_pregnancy8	0.0062	0.577	9
Active_smoke_pregnancy9	0.0055	0.705	10
Cig_pregnancy	0.0101	0.918	1
Cig_pregnancy1	0.0101	0.93	2
Cig_pregnancy10	0.0125	0.595	11
Cig_pregnancy11	0.0107	0.859	12
Cig_pregnancy12	0.0101	0.926	13
Cig_pregnancy13	0.0107	0.871	14
Cig_pregnancy14	0.0125	0.604	15
Cig_pregnancy15	0.0101	0.932	16
Cig_pregnancy16	0.0089	0.985	17
Cig_pregnancy17	0.0107	0.869	18
Cig_pregnancy18	0.0101	0.927	19
Cig_pregnancy19	0.0107	0.847	20
Cig_pregnancy2	0.0101	0.912	3
Cig_pregnancy3	0.0135	0.435	4
Cig_pregnancy4	0.0101	0.914	5

Cig_pregnancy5	0.0113	0.781	6
Cig_pregnancy6	0.0101	0.919	7
Cig_pregnancy7	0.0101	0.919	8
Cig_pregnancy8	0.0101	0.921	9
Cig_pregnancy9	0.0113	0.787	10
Cotinine_levels_4yrs	0.0075	0.276	1
Cotinine_levels_4yrs1	0.0070	0.361	2
Cotinine_levels_4yrs10	0.0072	0.323	11
Cotinine_levels_4yrs11	0.0074	0.302	12
Cotinine_levels_4yrs12	0.0069	0.372	13
Cotinine_levels_4yrs13	0.0077	0.247	14
Cotinine_levels_4yrs14	0.0073	0.302	15
Cotinine_levels_4yrs15	0.0075	0.254	16
Cotinine_levels_4yrs16	0.0065	0.485	17
Cotinine_levels_4yrs17	0.0066	0.476	18
Cotinine_levels_4yrs18	0.0067	0.416	19
Cotinine_levels_4yrs19	0.0070	0.332	20
Cotinine_levels_4yrs2	0.0073	0.309	3
Cotinine_levels_4yrs3	0.0075	0.269	4
Cotinine_levels_4yrs4	0.0068	0.426	5
Cotinine_levels_4yrs5	0.0065	0.473	6
Cotinine_levels_4yrs6	0.0076	0.251	7
Cotinine_levels_4yrs7	0.0075	0.26	8
Cotinine_levels_4yrs8	0.0074	0.315	9
Cotinine_levels_4yrs9	0.0069	0.406	10
Maternal_cotinine_levels	0.0046	0.894	1
Maternal_cotinine_levels1	0.0052	0.8	2
Maternal_cotinine_levels10	0.0056	0.7	11

Maternal_cotinine_levels11	0.0056	0.727	12
Maternal_cotinine_levels12	0.0055	0.734	13
Maternal_cotinine_levels13	0.0056	0.743	14
Maternal_cotinine_levels14	0.0055	0.746	15
Maternal_cotinine_levels15	0.0047	0.892	16
Maternal_cotinine_levels16	0.0046	0.923	17
Maternal_cotinine_levels17	0.0048	0.871	18
Maternal_cotinine_levels18	0.0048	0.869	19
Maternal_cotinine_levels19	0.0048	0.861	20
Maternal_cotinine_levels2	0.0046	0.906	3
Maternal_cotinine_levels3	0.0056	0.707	4
Maternal_cotinine_levels4	0.0047	0.9	5
Maternal_cotinine_levels5	0.0055	0.713	6
Maternal_cotinine_levels6	0.0046	0.895	7
Maternal_cotinine_levels7	0.0048	0.854	8
Maternal_cotinine_levels8	0.0047	0.894	9
Maternal_cotinine_levels9	0.0046	0.913	10
Maternal_smoking_dose_duration	0.0122	0.649	1
Maternal_smoking_dose_duration1	0.0120	0.706	2
Maternal_smoking_dose_duration10	0.0122	0.661	11
Maternal_smoking_dose_duration11	0.0129	0.512	12
Maternal_smoking_dose_duration12	0.0146	0.275	13
Maternal_smoking_dose_duration13	0.0113	0.769	14
Maternal_smoking_dose_duration14	0.0122	0.678	15
Maternal_smoking_dose_duration15	0.0123	0.618	16
Maternal_smoking_dose_duration16	0.0122	0.677	17
Maternal_smoking_dose_duration17	0.0110	0.825	18
Maternal_smoking_dose_duration18	0.0110	0.826	19

Maternal_smoking_dose_duration19	0.0122	0.671	20
Maternal_smoking_dose_duration2	0.0116	0.753	3
Maternal_smoking_dose_duration3	0.0122	0.656	4
Maternal_smoking_dose_duration4	0.0120	0.669	5
Maternal_smoking_dose_duration5	0.0129	0.504	6
Maternal_smoking_dose_duration6	0.0110	0.815	7
Maternal_smoking_dose_duration7	0.0110	0.831	8
Maternal_smoking_dose_duration8	0.0110	0.818	9
Maternal_smoking_dose_duration9	0.0110	0.831	10
Secondhandsmoke_all	0.0064	0.509	1
Secondhandsmoke_all_4yrs	0.0065	0.469	1
Secondhandsmoke_all_4yrs1	0.0065	0.475	2
Secondhandsmoke_all_4yrs10	0.0069	0.415	11
Secondhandsmoke_all_4yrs11	0.0070	0.37	12
Secondhandsmoke_all_4yrs12	0.0069	0.378	13
Secondhandsmoke_all_4yrs13	0.0069	0.401	14
Secondhandsmoke_all_4yrs14	0.0065	0.488	15
Secondhandsmoke_all_4yrs15	0.0065	0.476	16
Secondhandsmoke_all_4yrs16	0.0065	0.5	17
Secondhandsmoke_all_4yrs17	0.0069	0.374	18
Secondhandsmoke_all_4yrs18	0.0064	0.503	19
Secondhandsmoke_all_4yrs19	0.0063	0.536	20
Secondhandsmoke_all_4yrs2	0.0065	0.544	3
Secondhandsmoke_all_4yrs3	0.0072	0.343	4
Secondhandsmoke_all_4yrs4	0.0063	0.531	5
Secondhandsmoke_all_4yrs5	0.0067	0.454	6
Secondhandsmoke_all_4yrs6	0.0063	0.533	7
Secondhandsmoke_all_4yrs7	0.0070	0.375	8

Secondhandsmoke_all_4yrs8	0.0070	0.355	9
Secondhandsmoke_all_4yrs9	0.0070	0.371	10
Secondhandsmoke_all1	0.0060	0.628	2
Secondhandsmoke_all10	0.0064	0.498	11
Secondhandsmoke_all11	0.0070	0.366	12
Secondhandsmoke_all12	0.0066	0.427	13
Secondhandsmoke_all13	0.0070	0.373	14
Secondhandsmoke_all14	0.0063	0.523	15
Secondhandsmoke_all15	0.0066	0.471	16
Secondhandsmoke_all16	0.0060	0.623	17
Secondhandsmoke_all17	0.0064	0.505	18
Secondhandsmoke_all18	0.0070	0.375	19
Secondhandsmoke_all19	0.0069	0.42	20
Secondhandsmoke_all2	0.0066	0.448	3
Secondhandsmoke_all3	0.0069	0.398	4
Secondhandsmoke_all4	0.0063	0.548	5
Secondhandsmoke_all5	0.0066	0.458	6
Secondhandsmoke_all6	0.0066	0.428	7
Secondhandsmoke_all7	0.0066	0.476	8
Secondhandsmoke_all8	0.0069	0.363	9
Secondhandsmoke_all9	0.0066	0.469	10
Secondhandsmoke_home	0.0075	0.275	1
Secondhandsmoke_home_4yrs	0.0056	0.697	1
Secondhandsmoke_home_4yrs1	0.0063	0.492	2
Secondhandsmoke_home_4yrs10	0.0069	0.39	11
Secondhandsmoke_home_4yrs11	0.0062	0.522	12
Secondhandsmoke_home_4yrs12	0.0052	0.782	13
Secondhandsmoke_home_4yrs13	0.0060	0.613	14

Secondhandsmoke_home_4yrs14	0.0067	0.444	15
Secondhandsmoke_home_4yrs15	0.0065	0.482	16
Secondhandsmoke_home_4yrs16	0.0063	0.542	17
Secondhandsmoke_home_4yrs17	0.0058	0.654	18
Secondhandsmoke_home_4yrs18	0.0061	0.586	19
Secondhandsmoke_home_4yrs19	0.0062	0.557	20
Secondhandsmoke_home_4yrs2	0.0065	0.484	3
Secondhandsmoke_home_4yrs3	0.0065	0.492	4
Secondhandsmoke_home_4yrs4	0.0063	0.53	5
Secondhandsmoke_home_4yrs5	0.0060	0.618	6
Secondhandsmoke_home_4yrs6	0.0062	0.564	7
Secondhandsmoke_home_4yrs7	0.0064	0.539	8
Secondhandsmoke_home_4yrs8	0.0063	0.521	9
Secondhandsmoke_home_4yrs9	0.0067	0.455	10
Secondhandsmoke_home1	0.0075	0.258	2
Secondhandsmoke_home10	0.0079	0.231	11
Secondhandsmoke_home11	0.0077	0.235	12
Secondhandsmoke_home12	0.0075	0.281	13
Secondhandsmoke_home13	0.0069	0.421	14
Secondhandsmoke_home14	0.0077	0.225	15
Secondhandsmoke_home15	0.0075	0.261	16
Secondhandsmoke_home16	0.0069	0.405	17
Secondhandsmoke_home17	0.0069	0.4	18
Secondhandsmoke_home18	0.0073	0.294	19
Secondhandsmoke_home19	0.0077	0.246	20
Secondhandsmoke_home2	0.0075	0.274	3
Secondhandsmoke_home3	0.0077	0.244	4
Secondhandsmoke_home4	0.0075	0.265	5

Secondhandsmoke_home5	0.0077	0.241	6
Secondhandsmoke_home6	0.0077	0.239	7
Secondhandsmoke_home7	0.0073	0.316	8
Secondhandsmoke_home8	0.0071	0.356	9
Secondhandsmoke_home9	0.0073	0.321	10
SHSUrinaryCotinine_4yrs	0.0048	0.859	1
SHSUrinaryCotinine_4yrs1	0.0057	0.67	2
SHSUrinaryCotinine_4yrs10	0.0048	0.86	11
SHSUrinaryCotinine_4yrs11	0.0055	0.688	12
SHSUrinaryCotinine_4yrs12	0.0055	0.763	13
SHSUrinaryCotinine_4yrs13	0.0051	0.811	14
SHSUrinaryCotinine_4yrs14	0.0051	0.815	15
SHSUrinaryCotinine_4yrs15	0.0049	0.849	16
SHSUrinaryCotinine_4yrs16	0.0057	0.664	17
SHSUrinaryCotinine_4yrs17	0.0057	0.682	18
SHSUrinaryCotinine_4yrs18	0.0057	0.681	19
SHSUrinaryCotinine_4yrs19	0.0050	0.831	20
SHSUrinaryCotinine_4yrs2	0.0055	0.731	3
SHSUrinaryCotinine_4yrs3	0.0050	0.835	4
SHSUrinaryCotinine_4yrs4	0.0052	0.797	5
SHSUrinaryCotinine_4yrs5	0.0055	0.744	6
SHSUrinaryCotinine_4yrs6	0.0050	0.83	7
SHSUrinaryCotinine_4yrs7	0.0049	0.864	8
SHSUrinaryCotinine_4yrs8	0.0057	0.699	9
SHSUrinaryCotinine_4yrs9	0.0051	0.841	10
SHSUrinaryCotinine_Preg	0.0065	0.498	1
SHSUrinaryCotinine_Preg1	0.0076	0.238	2
SHSUrinaryCotinine_Preg10	0.0065	0.481	11

SHSUrinaryCotinine_Preg11	0.0069	0.391	12
SHSUrinaryCotinine_Preg12	0.0069	0.367	13
SHSUrinaryCotinine_Preg13	0.0065	0.491	14
SHSUrinaryCotinine_Preg14	0.0065	0.506	15
SHSUrinaryCotinine_Preg15	0.0072	0.334	16
SHSUrinaryCotinine_Preg16	0.0068	0.424	17
SHSUrinaryCotinine_Preg17	0.0065	0.45	18
SHSUrinaryCotinine_Preg18	0.0072	0.324	19
SHSUrinaryCotinine_Preg19	0.0065	0.492	20
SHSUrinaryCotinine_Preg2	0.0065	0.489	3
SHSUrinaryCotinine_Preg3	0.0065	0.488	4
SHSUrinaryCotinine_Preg4	0.0068	0.422	5
SHSUrinaryCotinine_Preg5	0.0069	0.385	6
SHSUrinaryCotinine_Preg6	0.0072	0.327	7
SHSUrinaryCotinine_Preg7	0.0065	0.487	8
SHSUrinaryCotinine_Preg8	0.0072	0.349	9
SHSUrinaryCotinine_Preg9	0.0078	0.225	10
Sustained_smoke_pregnancy	0.0050	0.841	1
Sustained_smoke_pregnancy1	0.0050	0.822	2
Sustained_smoke_pregnancy10	0.0068	0.404	11
Sustained_smoke_pregnancy11	0.0068	0.425	12
Sustained_smoke_pregnancy12	0.0068	0.413	13
Sustained_smoke_pregnancy13	0.0068	0.421	14
Sustained_smoke_pregnancy14	0.0068	0.416	15
Sustained_smoke_pregnancy15	0.0062	0.541	16
Sustained_smoke_pregnancy16	0.0068	0.437	17
Sustained_smoke_pregnancy17	0.0068	0.423	18
Sustained_smoke_pregnancy18	0.0046	0.925	19

Sustained_smoke_pregnancy19	0.0068	0.421	20
Sustained_smoke_pregnancy2	0.0068	0.428	3
Sustained_smoke_pregnancy3	0.0068	0.393	4
Sustained_smoke_pregnancy4	0.0050	0.83	5
Sustained_smoke_pregnancy5	0.0068	0.422	6
Sustained_smoke_pregnancy6	0.0069	0.377	7
Sustained_smoke_pregnancy7	0.0050	0.838	8
Sustained_smoke_pregnancy8	0.0069	0.383	9
Sustained_smoke_pregnancy9	0.0068	0.413	10

Supplementary Table S6. Association study of tobacco and mercury exposures from the prenatal to childhood period with the gut microbiome at a genus level, in an unadjusted and an adjusted multivariable association model (MaAsLin2 analysis). The best 10 results (genus) with the smallest *q*-values obtained for each individual pollutant exposure are shown. Exposures to tobacco (151 children) were adjusted by maternal education, maternal BMI and having siblings at birth (previously selected by the *envfit* function). Exposure to mercury during pregnancy (148 children) was adjusted by original data of fish intake at 32 weeks of pregnancy, maternal education and having siblings at birth. Exposure to mercury at 4 years (151 children) was adjusted by fish intake at 4 years, maternal education and having siblings at birth. Abbreviation: qByGenus, *q*-value obtained from the raw MaAsLin2 output table and calculated with the BH correction.

**UNADJUSTED MERCURY**						
**feature**	**metadata**	**value**	**coef**	**stderr**	**pval**	**qByGenus**
Flavonifractor	hg_imp_0	hg_imp_0	0.4062	0.1825	0.0279	0.8377
Intestinimonas	hg_imp_0	hg_imp_0	0.4875	0.2063	0.0198	0.8377
Fusicatenibacter	hg_imp_0	hg_imp_0	-0.3045	0.1891	0.1100	0.8655
Eisenbergiella	hg_imp_0	hg_imp_0	-0.2977	0.1850	0.1102	0.8655
Hungatella	hg_imp_0	hg_imp_0	-0.2854	0.1618	0.0802	0.8655
Oscillibacter	hg_imp_0	hg_imp_0	0.3344	0.2108	0.1154	0.8655
Colidextribacter	hg_imp_0	hg_imp_0	0.2851	0.1456	0.0525	0.8655
Bacteroides	hg_imp_0	hg_imp_0	0.1452	0.0834	0.0843	0.8655
Paludicola	hg_imp_0	hg_imp_0	0.2381	0.1769	0.1809	0.9044
Akkermansia	hg_imp_0	hg_imp_0	-0.6114	0.4527	0.1795	0.9044
CAG.56	hg_raw_4	hg_raw_4	0.3059	0.1577	0.0545	0.5638
UC5.1.2E3	hg_raw_4	hg_raw_4	-0.2302	0.1325	0.0846	0.5638
UBA1819	hg_raw_4	hg_raw_4	-0.2999	0.1420	0.0365	0.5638
Faecalibacterium	hg_raw_4	hg_raw_4	0.2393	0.1372	0.0834	0.5638
UCG.003	hg_raw_4	hg_raw_4	0.4017	0.2229	0.0737	0.5638
Akkermansia	hg_raw_4	hg_raw_4	-0.8556	0.4239	0.0455	0.5638
Erysipelatoclostridium	hg_raw_4	hg_raw_4	-0.4724	0.1891	0.0137	0.5638

Intestinibacter	hg_raw_4	hg_raw_4	-0.2924	0.1654	0.0793	0.5638
Clostridium_sensu_stricto_1	hg_raw_4	hg_raw_4	-0.5714	0.2538	0.0260	0.5638
Lachnospiraceae_UCG.004	hg_raw_4	hg_raw_4	0.2625	0.1580	0.0990	0.5938
**ADJUSTED MERCURY**						
**feature**	**metadata**	**value**	**coef**	**stderr**	**pval**	**qByGenus**
Intestinimonas	hg_imp_0	hg_imp_0	0.7105	0.2286	0.0024	0.1439
Oscillibacter	hg_imp_0	hg_imp_0	0.5449	0.2366	0.0232	0.6959
Fusicatenibacter	hg_imp_0	hg_imp_0	-0.4142	0.2153	0.0570	0.7942
Colidextribacter	hg_imp_0	hg_imp_0	0.2828	0.1673	0.0938	0.7942
Flavonifractor	hg_imp_0	hg_imp_0	0.3875	0.2090	0.0665	0.7942
Streptococcus	hg_imp_0	hg_imp_0	-0.3466	0.2059	0.0952	0.7942
Clostridium_sensu_stricto_1	hg_imp_0	hg_imp_0	-0.4981	0.3055	0.1059	0.7942
Parabacteroides	hg_imp_0	hg_imp_0	0.3355	0.2053	0.1051	0.7942
Agathobacter	hg_imp_0	hg_imp_0	-0.1190	0.1642	0.4702	0.9728
Roseburia	hg_imp_0	hg_imp_0	-0.1648	0.2033	0.4194	0.9272
Erysipelatoclostridium	hg_raw_4	hg_raw_4	-0.5533	0.2057	0.0081	0.4216
CAG.56	hg_raw_4	hg_raw_4	0.3856	0.1680	0.0233	0.4216
Faecalibacterium	hg_raw_4	hg_raw_4	0.3259	0.1459	0.0273	0.4216
UCG.002	hg_raw_4	hg_raw_4	0.4864	0.2370	0.0422	0.4216
UCG.003	hg_raw_4	hg_raw_4	0.5327	0.2402	0.0283	0.4216
Clostridium_sensu_stricto_1	hg_raw_4	hg_raw_4	-0.5675	0.2762	0.0419	0.4216
Roseburia	hg_raw_4	hg_raw_4	0.3185	0.1811	0.0811	0.6308
UBA1819	hg_raw_4	hg_raw_4	-0.2511	0.1515	0.1000	0.6308

Collinsella	hg_raw_4	hg_raw_4	-0.3573	0.2216	0.1093	0.6308
Dialister	hg_raw_4	hg_raw_4	0.7235	0.4446	0.1061	0.6308
**TOBACCO UNADJUSTED**						
**feature**	**metadata**	**value**	**coef**	**stderr**	**pval**	**qByGenus**
Akkermansia	msmk_anyt	yes	-3.7643	0.9188	0.0001	0.0042
Dorea	msmk_anyt	yes	1.0192	0.3527	0.0044	0.1333
Lachnoclostridium	msmk_anyt	yes	0.9977	0.4342	0.0230	0.3448
UBA1819	msmk_anyt	yes	0.6759	0.2934	0.0227	0.3448
Lachnospiraceae_NK4A136_group	msmk_anyt	yes	-0.7328	0.3490	0.0375	0.3509
Intestinimonas	msmk_anyt	yes	-0.8837	0.4407	0.0468	0.3509
Collinsella	msmk_anyt	yes	0.9210	0.4476	0.0414	0.3509
Haemophilus	msmk_anyt	yes	1.0826	0.5395	0.0466	0.3509
Escherichia.Shigella	msmk_anyt	yes	1.4711	0.7565	0.0537	0.3582
Roseburia	msmk_anyt	yes	-0.6843	0.3797	0.0736	0.3839
Dorea	msmk_sust	yes	2.0100	0.5049	0.0001	0.0065
UBA1819	msmk_sust	yes	1.1009	0.4284	0.0112	0.1676
Collinsella	msmk_sust	yes	1.7180	0.6502	0.0091	0.1676
Akkermansia	msmk_sust	yes	-3.6848	1.3893	0.0089	0.1676
Lachnospiraceae_NK4A136_group	msmk_sust	yes	-1.2197	0.5094	0.0179	0.1792
Blautia	msmk_sust	yes	1.1556	0.4774	0.0167	0.1792
Anaerostipes	msmk_sust	yes	1.5150	0.7087	0.0342	0.2931
Butyricimonas	msmk_sust	yes	1.7007	0.8749	0.0538	0.2978
UCG.002	msmk_sust	yes	-1.4396	0.7135	0.0455	0.2978

Colidextribacter	msmk_sust	yes	-0.8208	0.4324	0.0597	0.2978
Dorea	msmk_dose	non-sustained/low/high	1.7873	0.4788	0.0003	0.0326
Incertae_Sedis	msmk_dose	only passive	-1.0603	0.3756	0.0054	0.1678
Collinsella	msmk_dose	non-sustained/low/high	1.6431	0.6194	0.0089	0.1678
Akkermansia	msmk_dose	non-sustained/low/high	-3.6260	1.3417	0.0077	0.1678
Turicibacter	msmk_dose	only passive	-1.3864	0.4963	0.0059	0.1678
Clostridium_sensu_stricto_1	msmk_dose	only passive	-1.4890	0.5457	0.0071	0.1678
Bacteroides	msmk_dose	only passive	0.4462	0.1704	0.0098	0.1678
Lachnospiraceae_NK4A136_group	msmk_dose	non-sustained/low/high	-1.1954	0.4922	0.0164	0.2458
Roseburia	msmk_dose	non-sustained/low/high	-1.2171	0.5322	0.0237	0.2581
Barnesiella	msmk_dose	only passive	-2.3809	1.0259	0.0217	0.2581
UBA1819	shs_global_4	yes	0.8375	0.2681	0.0022	0.1303
Phascolarctobacterium	shs_global_4	yes	1.8122	0.6984	0.0105	0.3148
Flavonifractor	shs_global_4	yes	0.7638	0.3425	0.0273	0.3696
NK4A214_group	shs_global_4	yes	-1.0050	0.4506	0.0273	0.3696
Dorea	shs_global_4	yes	0.6875	0.3151	0.0308	0.3696
Bilophila	shs_global_4	yes	0.7394	0.3627	0.0434	0.4341
Roseburia	shs_global_4	yes	-0.4750	0.3479	0.1744	0.6848
UC5.1.2E3	shs_global_4	yes	0.4727	0.2912	0.1068	0.6848
Faecalibacterium	shs_global_4	yes	0.4211	0.2850	0.1418	0.6848
Oscillibacter	shs_global_4	yes	0.6619	0.3968	0.0976	0.6848
Agathobacter	shs_global_7	yes	0.2630	0.3023	0.3858	0.9249
Roseburia	shs_global_7	yes	-0.4475	0.3682	0.2261	0.9249

CAG.56	shs_global_7	yes	0.3340	0.3416	0.3298	0.9249
Fusicatenibacter	shs_global_7	yes	-0.3202	0.3677	0.3852	0.9249
Eisenbergiella	shs_global_7	yes	0.3024	0.3498	0.3888	0.9249
Lachnospiraceae_UCG.004	shs_global_7	yes	0.5884	0.3351	0.0812	0.9249
Lachnospiraceae_NK4A136_group	shs_global_7	yes	-0.1933	0.3338	0.5634	0.9249
Lachnoclostridium	shs_global_7	yes	0.8004	0.4216	0.0596	0.9249
Barnesiella	shs_global_7	yes	1.1677	0.9896	0.2399	0.9249
Odoribacter	shs_global_7	yes	0.2386	0.3283	0.4684	0.9249
Dorea	shs_indoors_4	yes	0.9283	0.3390	0.0070	0.4221
Agathobacter	shs_indoors_4	yes	0.3449	0.3120	0.2710	0.8749
Roseburia	shs_indoors_4	yes	-0.5829	0.3727	0.1202	0.8749
CAG.56	shs_indoors_4	yes	-0.3803	0.3490	0.2778	0.8749
UC5.1.2E3	shs_indoors_4	yes	0.3217	0.3149	0.3088	0.8749
Hungatella	shs_indoors_4	yes	-0.4648	0.3187	0.1470	0.8749
Lachnospiraceae_NK4A136_group	shs_indoors_4	yes	-0.3987	0.3443	0.2490	0.8749
UBA1819	shs_indoors_4	yes	0.5199	0.3092	0.0950	0.8749
Paludicola	shs_indoors_4	yes	-0.4472	0.3681	0.2266	0.8749
Colidextribacter	shs_indoors_4	yes	-0.3555	0.2888	0.2205	0.8749
Lachnoclostridium	shs_indoors_7	yes	0.9588	0.4717	0.0439	0.6612
Flavonifractor	shs_indoors_7	yes	0.7517	0.4060	0.0661	0.6612
Streptococcus	shs_indoors_7	yes	0.8226	0.4268	0.0559	0.6612
Dorea	shs_indoors_7	yes	0.7227	0.3854	0.0628	0.6612
Lachnospiraceae_UCG.001	shs_indoors_7	yes	-0.7730	0.3820	0.0448	0.6612

Coprococcus	shs_indoors_7	yes	-0.9092	0.4839	0.0622	0.6612
Roseburia	shs_indoors_7	yes	-0.6311	0.4114	0.1273	0.7123
Eisenbergiella	shs_indoors_7	yes	0.6105	0.3898	0.1196	0.7123
Subdoligranulum	shs_indoors_7	yes	-0.6646	0.4451	0.1376	0.7123
Butyricicoccus	shs_indoors_7	yes	0.8234	0.5583	0.1425	0.7123
UBA1819	ctabpreg	ctabpreg	0.3577	0.1251	0.0049	0.2927
Roseburia	ctabpreg	ctabpreg	-0.3045	0.1633	0.0643	0.3671
Eisenbergiella	ctabpreg	ctabpreg	0.3012	0.1560	0.0555	0.3671
Barnesiella	ctabpreg	ctabpreg	1.1059	0.4419	0.0134	0.3671
Butyricimonas	ctabpreg	ctabpreg	0.4865	0.2571	0.0604	0.3671
Butyricicoccus	ctabpreg	ctabpreg	-0.4146	0.2271	0.0700	0.3671
Collinsella	ctabpreg	ctabpreg	0.3803	0.1929	0.0505	0.3671
Escherichia.Shigella	ctabpreg	ctabpreg	0.7460	0.3240	0.0227	0.3671
Akkermansia	ctabpreg	ctabpreg	-0.7449	0.4131	0.0734	0.3671
Phascolarctobacterium	ctabpreg	ctabpreg	0.6636	0.3355	0.0498	0.3671
UBA1819	crcotinine_0	crcotinine_0	0.3689	0.1302	0.0053	0.3166
Escherichia.Shigella	crcotinine_0	crcotinine_0	0.8327	0.3221	0.0107	0.3218
Lachnospiraceae_NK4A136_group	crcotinine_0	crcotinine_0	-0.2361	0.1505	0.1188	0.6792
Barnesiella	crcotinine_0	crcotinine_0	0.5451	0.4463	0.2239	0.6792
Butyricimonas	crcotinine_0	crcotinine_0	0.4912	0.2549	0.0559	0.6792
Subdoligranulum	crcotinine_0	crcotinine_0	-0.2093	0.1657	0.2085	0.6792
UCG.003	crcotinine_0	crcotinine_0	-0.2830	0.2123	0.1845	0.6792
Colidextribacter	crcotinine_0	crcotinine_0	-0.2624	0.1270	0.0406	0.6792

Flavonifractor	crcotinine_0	crcotinine_0	-0.2204	0.1664	0.1874	0.6792
Bifidobacterium	crcotinine_0	crcotinine_0	0.2308	0.1925	0.2327	0.6792
Akkermansia	crcotinine_4	crcotinine_4	-0.8672	0.4113	0.0368	0.5513
Streptococcus	crcotinine_4	crcotinine_4	-0.3641	0.1711	0.0350	0.5513
Christensenellaceae_R.7_group	crcotinine_4	crcotinine_4	-0.5063	0.2027	0.0136	0.5513
Blautia	crcotinine_4	crcotinine_4	-0.3236	0.1418	0.0239	0.5513
NK4A214_group	crcotinine_4	crcotinine_4	-0.4033	0.2172	0.0654	0.7852
CAG.56	crcotinine_4	crcotinine_4	-0.2018	0.1552	0.1957	0.8461
Fusicatenibacter	crcotinine_4	crcotinine_4	-0.2205	0.1674	0.1897	0.8461
Subdoligranulum	crcotinine_4	crcotinine_4	-0.2603	0.1806	0.1518	0.8461
UBA1819	crcotinine_4	crcotinine_4	0.1698	0.1374	0.2187	0.8461
Paludicola	crcotinine_4	crcotinine_4	-0.2230	0.1667	0.1831	0.8461
UBA1819	cotinine_0cat	exposed	0.7290	0.2603	0.0058	0.2657
Dialister	cotinine_0cat	exposed	-2.0753	0.7822	0.0089	0.2657
Odoribacter	cotinine_0cat	exposed	0.6837	0.2973	0.0229	0.4579
Anaerostipes	cotinine_0cat	exposed	-0.8113	0.4168	0.0535	0.8029
Parasutterella	cotinine_0cat	exposed	-0.7603	0.4393	0.0857	0.8566
Dorea	cotinine_0cat	exposed	0.5459	0.3135	0.0838	0.8566
Agathobacter	cotinine_0cat	exposed	0.2125	0.2751	0.4411	0.9463
Roseburia	cotinine_0cat	exposed	-0.4079	0.3237	0.2096	0.9463
CAG.56	cotinine_0cat	exposed	-0.4192	0.3058	0.1725	0.9463
Fusicatenibacter	cotinine_0cat	exposed	0.2909	0.3380	0.3910	0.9463
Roseburia	cotinine_4cat	exposed	-0.5432	0.3638	0.1376	0.6054

Barnesiella	cotinine_4cat	exposed	-1.5370	0.9852	0.1209	0.6054
Paludicola	cotinine_4cat	exposed	-0.8957	0.3558	0.0129	0.6054
UCG.002	cotinine_4cat	exposed	-0.6818	0.4755	0.1538	0.6054
Colidextribacter	cotinine_4cat	exposed	-0.4394	0.2726	0.1092	0.6054
NK4A214_group	cotinine_4cat	exposed	-0.7420	0.4724	0.1185	0.6054
Bifidobacterium	cotinine_4cat	exposed	0.7306	0.4184	0.0829	0.6054
Collinsella	cotinine_4cat	exposed	0.5996	0.4245	0.1600	0.6054
Akkermansia	cotinine_4cat	exposed	-2.0351	0.8892	0.0236	0.6054
Phascolarctobacterium	cotinine_4cat	exposed	1.5194	0.7371	0.0411	0.6054
**TOBACCO ADJUSTED**						
**feature**	**metadata**	**value**	**coef**	**stderr**	**pval**	**qByGenus**
Akkermansia	msmk_anyt	yes	-3.7498	0.9279	0.0001	0.0052
Dorea	msmk_anyt	yes	1.0710	0.3776	0.0052	0.1571
Lachnoclostridium	msmk_anyt	yes	1.1876	0.4613	0.0111	0.2218
Lachnospiraceae_NK4A136_group	msmk_anyt	yes	-0.7343	0.3754	0.0525	0.4497
UBA1819	msmk_anyt	yes	0.6379	0.3056	0.0387	0.4497
UCG.005	msmk_anyt	yes	-1.0688	0.5307	0.0459	0.4497
Christensenellaceae_R.7_group	msmk_anyt	yes	-0.9768	0.4755	0.0418	0.4497
Collinsella	msmk_anyt	yes	0.8927	0.4769	0.0633	0.4747
Roseburia	msmk_anyt	yes	-0.4884	0.4047	0.2295	0.5464
Butyricimonas	msmk_anyt	yes	0.7966	0.6405	0.2157	0.5464
Dorea	msmk_sust	yes	2.0427	0.5279	0.0002	0.0100
Akkermansia	msmk_sust	yes	-4.1027	1.3590	0.0030	0.0905

Collinsella	msmk_sust	yes	1.7957	0.6737	0.0086	0.1259
Blautia	msmk_sust	yes	1.2883	0.4966	0.0105	0.1259
Anaerostipes	msmk_sust	yes	1.9106	0.7124	0.0082	0.1259
Lachnospiraceae_NK4A136_group	msmk_sust	yes	-1.3108	0.5329	0.0151	0.1513
UBA1819	msmk_sust	yes	1.0178	0.4355	0.0208	0.1787
UCG.002	msmk_sust	yes	-1.6636	0.7315	0.0245	0.1836
Butyricimonas	msmk_sust	yes	1.8643	0.9077	0.0418	0.2789
Paludicola	msmk_sust	yes	-1.1349	0.5788	0.0519	0.3114
Akkermansia	msmk_dose	non-sustained/low/high	-4.2629	1.3097	0.0014	0.0855
Dorea	msmk_dose	non-sustained/low/high	1.7349	0.4992	0.0007	0.0817
Lachnospiraceae_NK4A136_group	msmk_dose	non-sustained/low/high	-1.2688	0.5162	0.0152	0.2280
Incertae_Sedis	msmk_dose	only passive	-1.0145	0.3915	0.0106	0.2117
Collinsella	msmk_dose	non-sustained/low/high	1.5922	0.6407	0.0141	0.2280
Turicibacter	msmk_dose	only passive	-1.3983	0.5204	0.0081	0.2117
Clostridium_sensu_stricto_1	msmk_dose	only passive	-1.5318	0.5698	0.0081	0.2117
Bacteroides	msmk_dose	only passive	0.4520	0.1739	0.0103	0.2117
Barnesiella	msmk_dose	only passive	-2.3795	1.0676	0.0274	0.2831
UCG.002	msmk_dose	non-sustained/low/high	-1.5675	0.7073	0.0283	0.2831
Flavonifractor	shs_global_4	yes	1.0207	0.3674	0.0063	0.2105
NK4A214_group	shs_global_4	yes	-1.2646	0.4873	0.0105	0.2105
Phascolarctobacterium	shs_global_4	yes	1.9984	0.7574	0.0093	0.2105
UBA1819	shs_global_4	yes	0.6758	0.2902	0.0214	0.3207
Eisenbergiella	shs_global_4	yes	-0.5102	0.3659	0.1656	0.7804

Lachnospiraceae_UCG.004	shs_global_4	yes	0.5125	0.3453	0.1401	0.7804
Hungatella	shs_global_4	yes	-0.3365	0.3267	0.3049	0.7804
Lachnoclostridium	shs_global_4	yes	0.5521	0.4458	0.2178	0.7804
Barnesiella	shs_global_4	yes	1.2452	1.0243	0.2263	0.7804
Faecalibacterium	shs_global_4	yes	0.4090	0.3139	0.1948	0.7804
Lachnospiraceae_UCG.004	shs_global_7	yes	0.6370	0.3357	0.0599	0.8568
Lachnoclostridium	shs_global_7	yes	0.8254	0.4329	0.0586	0.8568
Dorea	shs_global_7	yes	0.6183	0.3523	0.0815	0.8568
Coprococcus	shs_global_7	yes	-0.7999	0.4360	0.0687	0.8568
Roseburia	shs_global_7	yes	-0.3890	0.3731	0.2989	0.8568
CAG.56	shs_global_7	yes	0.3563	0.3441	0.3023	0.8568
Fusicatenibacter	shs_global_7	yes	-0.3658	0.3762	0.3325	0.8568
Eisenbergiella	shs_global_7	yes	0.2915	0.3583	0.4172	0.8568
Barnesiella	shs_global_7	yes	1.3704	1.0058	0.1752	0.8568
Odoribacter	shs_global_7	yes	0.2679	0.3350	0.4253	0.8568
Roseburia	shs_indoors_4	yes	-0.4506	0.4050	0.2680	0.7674
CAG.56	shs_indoors_4	yes	-0.5453	0.3730	0.1463	0.7674
Hungatella	shs_indoors_4	yes	-0.7114	0.3430	0.0401	0.7674
Lachnospiraceae_NK4A136_group	shs_indoors_4	yes	-0.4680	0.3763	0.2160	0.7674
Paludicola	shs_indoors_4	yes	-0.6679	0.3952	0.0935	0.7674
UCG.002	shs_indoors_4	yes	-0.5848	0.4905	0.2354	0.7674
Colidextribacter	shs_indoors_4	yes	-0.3397	0.3140	0.2813	0.7674
Flavonifractor	shs_indoors_4	yes	0.5873	0.4038	0.1483	0.7674

NK4A214_group	shs_indoors_4	yes	-1.1920	0.5289	0.0259	0.7674
Butyricicoccus	shs_indoors_4	yes	0.6161	0.5636	0.2764	0.7674
Flavonifractor	shs_indoors_7	yes	0.9595	0.4056	0.0194	0.5400
Coprococcus	shs_indoors_7	yes	-1.1210	0.4925	0.0244	0.5400
Lachnoclostridium	shs_indoors_7	yes	1.0058	0.4912	0.0425	0.5400
UCG.005	shs_indoors_7	yes	-1.0623	0.5650	0.0622	0.5400
Streptococcus	shs_indoors_7	yes	0.8105	0.4324	0.0630	0.5400
Christensenellaceae_R.7_group	shs_indoors_7	yes	-1.0207	0.5137	0.0489	0.5400
Lachnospiraceae_UCG.001	shs_indoors_7	yes	-0.8474	0.3950	0.0337	0.5400
Roseburia	shs_indoors_7	yes	-0.5571	0.4232	0.1902	0.6377
CAG.56	shs_indoors_7	yes	-0.4024	0.3912	0.3054	0.7968
Fusicatenibacter	shs_indoors_7	yes	-0.5024	0.4270	0.2413	0.7620
Barnesiella	ctabpreg	ctabpreg	1.2181	0.4500	0.0076	0.3186
UBA1819	ctabpreg	ctabpreg	0.3249	0.1255	0.0106	0.3186
Akkermansia	ctabpreg	ctabpreg	-0.9081	0.3985	0.0242	0.3242
Escherichia.Shigella	ctabpreg	ctabpreg	0.7225	0.3276	0.0290	0.3242
Eisenbergiella	ctabpreg	ctabpreg	0.3159	0.1600	0.0504	0.3242
Butyricimonas	ctabpreg	ctabpreg	0.5082	0.2631	0.0554	0.3242
UCG.003	ctabpreg	ctabpreg	-0.4004	0.2107	0.0594	0.3242
Butyricicoccus	ctabpreg	ctabpreg	-0.4473	0.2314	0.0552	0.3242
Phascolarctobacterium	ctabpreg	ctabpreg	0.6544	0.3401	0.0563	0.3242
Dialister	ctabpreg	ctabpreg	-0.8317	0.4012	0.0400	0.3242
Escherichia.Shigella	crcotinine_0	crcotinine_0	0.8634	0.3310	0.0101	0.4507

UBA1819	crcotinine_0	crcotinine_0	0.3272	0.1329	0.0150	0.4507
Agathobacter	crcotinine_0	crcotinine_0	-0.1262	0.1390	0.3654	0.7284
CAG.56	crcotinine_0	crcotinine_0	-0.1671	0.1578	0.2914	0.7284
UC5.1.2E3	crcotinine_0	crcotinine_0	-0.1234	0.1437	0.3919	0.7284
Lachnospiraceae_NK4A136_group	crcotinine_0	crcotinine_0	-0.2634	0.1582	0.0981	0.6447
Barnesiella	crcotinine_0	crcotinine_0	0.7418	0.4627	0.1111	0.6447
Butyricimonas	crcotinine_0	crcotinine_0	0.5458	0.2650	0.0413	0.6447
Subdoligranulum	crcotinine_0	crcotinine_0	-0.2258	0.1745	0.1977	0.7284
Faecalibacterium	crcotinine_0	crcotinine_0	-0.1233	0.1401	0.3805	0.7284
Christensenellaceae_R.7_group	crcotinine_4	crcotinine_4	-0.5653	0.2044	0.0065	0.3618
Akkermansia	crcotinine_4	crcotinine_4	-0.9125	0.4001	0.0241	0.3618
Streptococcus	crcotinine_4	crcotinine_4	-0.3890	0.1702	0.0238	0.3618
Blautia	crcotinine_4	crcotinine_4	-0.3330	0.1451	0.0233	0.3618
NK4A214_group	crcotinine_4	crcotinine_4	-0.4443	0.2171	0.0427	0.5119
Oscillibacter	crcotinine_4	crcotinine_4	0.3257	0.1920	0.0921	0.8350
CAG.56	crcotinine_4	crcotinine_4	-0.2168	0.1560	0.1670	0.8350
Fusicatenibacter	crcotinine_4	crcotinine_4	-0.2385	0.1710	0.1653	0.8350
Subdoligranulum	crcotinine_4	crcotinine_4	-0.2700	0.1866	0.1502	0.8350
Paludicola	crcotinine_4	crcotinine_4	-0.2497	0.1708	0.1461	0.8350
Odoribacter	cotinine_0cat	exposed	0.7205	0.3092	0.0212	0.4860
UBA1819	cotinine_0cat	exposed	0.6271	0.2659	0.0198	0.4860
Dialister	cotinine_0cat	exposed	-1.8146	0.8125	0.0271	0.4860
Anaerostipes	cotinine_0cat	exposed	-0.9146	0.4233	0.0324	0.4860

Roseburia	cotinine_0cat	exposed	-0.3019	0.3374	0.3724	0.9027
CAG.56	cotinine_0cat	exposed	-0.5110	0.3135	0.1053	0.9027
Fusicatenibacter	cotinine_0cat	exposed	0.3204	0.3536	0.3664	0.9027
Eisenbergiella	cotinine_0cat	exposed	0.4055	0.3281	0.2185	0.9027
Hungatella	cotinine_0cat	exposed	-0.3379	0.2936	0.2518	0.9027
Lachnospiraceae_NK4A136_group	cotinine_0cat	exposed	-0.3175	0.3180	0.3198	0.9027
Paludicola	cotinine_4cat	exposed	-1.1252	0.3883	0.0044	0.2627
Akkermansia	cotinine_4cat	exposed	-1.9256	0.9329	0.0409	0.6310
Christensenellaceae_R.7_group	cotinine_4cat	exposed	-0.9823	0.4808	0.0430	0.6310
Alistipes	cotinine_4cat	exposed	-0.6783	0.3340	0.0442	0.6310
NK4A214_group	cotinine_4cat	exposed	-0.9019	0.5064	0.0771	0.6310
Bifidobacterium	cotinine_4cat	exposed	0.8377	0.4527	0.0664	0.6310
Phascolarctobacterium	cotinine_4cat	exposed	1.4492	0.8000	0.0723	0.6310
Hungatella	cotinine_4cat	exposed	-0.5626	0.3401	0.1003	0.6310
Collinsella	cotinine_4cat	exposed	0.7311	0.4618	0.1157	0.6310
Lachnospiraceae_UCG.001	cotinine_4cat	exposed	-0.5756	0.3583	0.1105	0.6310
